# Investigation of cholinesterase and α-glucosidase enzyme activities, and molecular docking and dft studies for 1,2-disubstituted cyclopentane derivatives with phenyl and benzyl units

**DOI:** 10.1007/s11030-024-10911-y

**Published:** 2024-07-08

**Authors:** Tekin Artunç, Yasin Çetinkaya, Parham Taslimi, Abdullah Menzek

**Affiliations:** 1 Department of Chemistry, Faculty of Science, Atatürk University, 25240 Erzurum, Turkey; 2 Department of Biotechnology, Faculty of Science, Bartin University, 74100 Bartin, Turkey; 3 Department of Emergency Aid and Disaster Management, Faculty of Health Sciences, Ardahan University, 75002 Ardahan, Turkey

**Keywords:** Bromination, α-Glucosidase, AChE, BChE, Density functional theory

## Abstract

Six known products (**4**–**9**) were prepared from reaction of adipoyl chloride with 1,2,3-trimethoxybenzene according to the literature. From (2,3,4-trimethoxyphenyl)(2-(2,3,4-trimethoxyphenyl)cyclopent-1-en-1-yl)methanone (**4**) of them, four new 1,2-disubstituted cyclopentane derivatives (**10–13**) with phenyl and benzyl units were synthesized by reactions such as hydrazonation, catalytic hydrogenation and bromination. The obtained compounds **4–13** were examined for their in vitro inhibitory activity against acetylcholinesterase (AChE), butyrylcholinesterase (BChE) and α-glucosidase enzymes. All compounds **4–13** showed inhibition at nanomolar level with *K*_*i*_ values in the range of 45.53 ± 7.35–631.96 ± 18.88 nM for AChE, 84.30 ± 9.92–622.10 ± 35.14 nM for BChE, and 25.47 ± 4.46–48.87 ± 7.33 for α-Glu. In silico molecular docking studies of the potent compounds were performed in the active sites of AChE (PDB: 1E66), BChE (PDB: 1P0I), and α-glucosidase (PDB: 5ZCC) to compare the effect of bromine atom on the inhibition mechanism. The optimized molecular structures, HOMO–LUMO energies and molecular electrostatic potential maps for the compounds were calculated by using density functional theory with B3LYP/6–31 + G(d,p).

**Supplementary Information:**

The online version contains supplementary material available at 10.1007/s11030-024-10911-y.

## Introduction

**Fig. 1:**
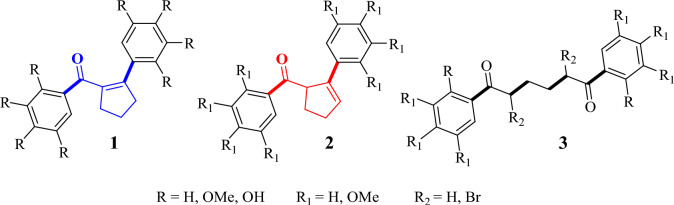
Some compounds formed by the reaction of benzene derivatives with adipoyl chloride

Phenol derivatives such as bromophenols exhibit biological activities such as antioxidant, carbonic anhydrase, α-glucosidase, α-amylase, and aldose reductase inhibitory activities. There are natural products in the phenol and bromophenol classes and they also show important biological activity [1–6]. In addition to the biological activities of these types of compounds, since they have electron-rich aromatic rings, they undergo reactions, especially electrophilic aromatic substitution reactions, and new derivatives are obtained. The structures and properties of these types of compounds are also studied mechanistically and theoretically*.* At different times before, we synthesized compounds with **1**–**3** structures formed by the reaction of benzene derivatives with methoxy groups at different positions with adipoyl chloride (Fig. 1). These compounds were published along with their biological properties [3, 7–9].

Alzheimer's disease (AD) is a neurological condition that affects older adults and is irreversible. The illness results in daily cognitive impairment, such as dementia and memory loss. The overproduction of the β-amyloid peptide, environmental variables, reactive oxygen species, and cholinergic insufficiency are some of the elements that affect how AD develops [10, 11]. Additionally, some metabolic enzymes, such as glycogen synthase kinase 3, acetylcholinesterase (AChE), butyrylcholinesterase (BChE), secretase, and cyclin-dependent kinase 5, as well as the N-methyl-D-aspartate receptor, play a key role in the progression of this disease. Cholinesterase inhibitors make up the majority of anti-Alzheimer's disease (AD) medications currently approved by the US Food and Drug Administration (FDA) [12]. The amount of the neurotransmitter acetylcholine in the brain is decreased as a result of cholinesterase enzymes (ChEs) hydrolyzing it more quickly. Cholinesterase inhibition thereby improves cholinergic neurotransmission.

The metabolism of carbohydrates depends on the enzyme α-glucosidase. By transforming starch and disaccharides into soluble monosaccharides like glucose, it elevates blood glucose levels in animals. Because of this activity, α-glucosidase has been suggested as a potential therapeutic target for the treatment of type-2 diabetes in people. Inhibition of this enzyme may reduce postprandial plasma glucose levels by delaying the digestion of carbohydrates and consequently the absorption of monosaccharides [13, 14]. Voglibose, acarbose, and miglitol, three often prescribed α-glucosidase inhibitors, have been linked to adverse effects include bloating, flatulence, diarrhea, soreness, and abdominal discomfort. Recently, it was discovered that bromophenols from marine algae serve as both protein tyrosine phosphatase (PTP1B) and α-glucosidase inhibitors [15–17].

**Fig. 2:**
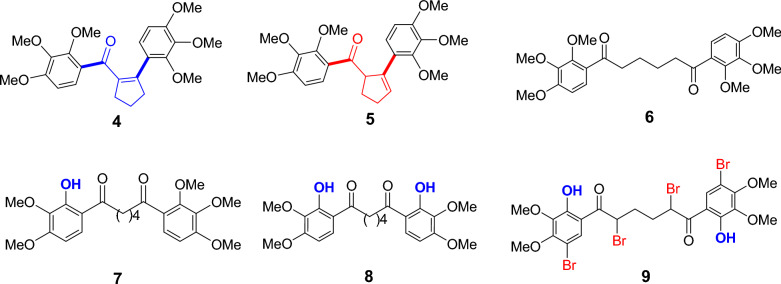
Compounds **4–9** synthesized in our previous study [7]

In our previous studies, we synthesized some derivatives of compounds **1**–**3**, and reported their biological activities such as carbonic anhydrase [3] and antioxidant [8]. In one of our recent articles [7], compounds **4–9** with OMe groups at positions 2, 3, and 4 on the phenyl rings of structures **1–3** were also obtained from the reaction of adipoyl chloride with 1,2,3-trimethoxybenzene (Fig. 2). In the study, DFT calculations including two hybrid functionals, B3LYP and M06-2X, for compounds **4**–**9** were performed [7]. The important experimental findings obtained in the ^1^H and ^13^C NMR data were reported by comparing them with computational data. Among compounds **4**–**9**, (2,3,4-trimethoxyphenyl)(2-(2,3,4-trimethoxyphenyl)cyclopent-1-ene-1-yl)methanone (**4**) and its derivative **5** are an important compound because they have different functional groups. For further reactions, the compound **4** was chosen due to its higher proportion than compound **5** in the reaction. Therefore, reactions such as hydrazonation, catalytic hydrogenation, and bromination reactions of **4** may be formed important products because the groups in the molecule, especially the non-aromatic double bonds, can interact and compounds with different functional groups can be formed. For this purpose, the reactions of **4** have been performed, and four new products have been obtained from these reactions. Moreover, DFT studies were investigated for four new products **10**–**13** to see if there was a connection between the properties of the compounds. In vitro inhibitory activities against AChE, α-glucosidase, and BChE have been investigated for compounds **4**–**13** because phenol and phenol derivative compounds show biological activity. In silico studies have been carried out for the most biologically active compounds. Also, when we examine the kinds of literature [18–20], there are studies similar to the compounds that we studied, so we examined the effects of novel compounds on some metabolic enzymes and added the results of the best enzymes to our study and then proved the results with another parameter such as molecular docking. For example, In the study of Bayrak et al. [20] every synthetic substance showed a significant inhibitory effect on both cholinergic enzymes. Lineweaver–Burk graphs were created in order to determine the Ki values of novel bromophenols. The range of Ki values for AChE, BChE, and α-glycosidase was determined to be 0.13–14.74 nM, 5.11–23.95 nM, and 63.96–206.78 nM, respectively. When compared to positive controls, all bromophenols and their derivatives show an effective inhibitory profile.

## Experimental section

### General information

The values of all chemicals, solvents, and silica gels (for column and thin layer chromatography), as well as measurements of Mp of all compounds used in this work, were obtained as described previously [7, 8].

### Synthesis

Synthesis of compounds **4–9** was given in the literature [7].

#### Synthesis of compound *10*

Similar to the literature [3, 21], to a solution of compound** 4** (0.1 g, 0.23 mmol) in acetic acid (15 mL) was added hydrazine hydrate (180 mg, 5.63 mmol) and the mixture was refluxed for 2 h. As a result of TLC, it was observed that the reaction was completed. After the reaction mixture cooled, it was poured into a mixture of water and ice (150 g). The mixture was extracted by CH_2_Cl_2_ (3 × 50 mL). Combined organic phases were dried over Na_2_SO_4_ and filtered and the solvent was removed in the evaporator. The residue was submitted to silica gel (20 g) column chromatography with EtOAc/hexane (2/3) elution. Product **10** (60 mg, 53%, pale yellow liquid) was obtained.

##### (Z)-N'-((2,3,4-Trimethoxyphenyl)(2-(2,3,4-trimethoxyphenyl)cyclopent-1-en-1-yl)methylene)acetohydrazide (10)

^1^H NMR (400 MHz—CDCl_3_): 8.20 (s, 1 NH), 6.49 (d, A part of system AB,* J* = 8.50 Hz, aromatic, 1H), 6.48 (d, A part of system AB,* J* = 8.50 Hz, aromatic, 1H), 6.36 (d, B part of system AB,* J* = 8.50 Hz, aromatic, 1H), 6.31 (d, B part of system AB,* J* = 8.50 Hz, aromatic, 1H), 3.83 (s, OCH_3_, 3H), 3.80 (s, OCH_3_, 3H), 3.76 (s, OCH_3_, 6H), 3.75 (s, OCH_3_, 6H), 2.89 (t, *J* = 7.60 Hz, aliphatic, 2H), 2.76 (t, *J* = 7.20 Hz, aliphatic, 2H), 2.21 (s, CH_3_, 3H), 2.04–1.95 (m, aliphatic, 2H); ^13^C NMR (100 MHz, CDCl_3_): 172.7 (CO), 154.5 (C=N), 152.4 (C), 150.5 (C), 150.3 (C), 147.2 (C), 143.8 (C), 141.7 (C), 141.4 (C), 136.8 (C), 125.4 (C), 124.0 (CH), 123.8 (CH), 118.0 (C), 107.3 (CH), 106.5 (CH), 60.8 (OCH_3_), 60.7 (OCH_3_), 60.6 (OCH_3_), 60.5 (OCH_3_), 56.0 (OCH_3_), 55.96 (OCH_3_), 41.2 (CH_2_), 35.3 (CH_2_), 22.0 (CH_2_), 20.4 (CH_3_); IR (CH_2_Cl_2_, cm^−1^): 3314, 2936, 2842, 1680, 1597, 1494, 1464, 1412, 1363, 1320, 1294, 1234, 1211, 1169, 1100, 1056, 1016, 984, 947, 914, 875, 796, 735, 691, 619, 553, 534. HRMS (ESI) m/z: [M + H]^+^ Calcd for C_26_H_33_N_2_O_7_ 485.2288; Found 485.2281.

#### Synthesis of compound *11*

A solution of compound **4** (90 mg, 0.21 mmol) in CH_2_Cl_2_ (20 mL) at room temperature in the presence of Pd/C was prepared according to the known catalytic hydrogenation procedure [3, 9, 22–24]. The reaction of this solution with hydrogen started and it was completed within 4 h according to thin layer chromatography (TLC). After the catalyst was removed by filtration and the solvent in the evaporator, product **11** (85 mg, 97%, colorless liquid) was obtained as the single product.

##### 1,2,3-Trimethoxy-4-(2-(2,3,4-trimethoxybenzyl)cyclopentyl)benzene (11)

^1^H NMR (400 MHz, CDCl_3_): 6.92 (d, A part of system AB,* J* = 8.40 Hz, aromatic, 1H), 6.66 (d, A part of system AB,* J* = 8.48 Hz, aromatic, 1H), 6.64 (d, B part of system AB, *J* = 8.65 Hz, aromatic, 1H), 6.52 (d, B part of system AB, *J* = 8.40 Hz, aromatic, 1H), 3.91 (s, OCH_3_, 3H), 3.87 (s, OCH_3_, 3H), 3.85 (s, OCH_3_, 3H), 3.82 (s, OCH_3_, 3H), 3.80 (s, OCH_3_, 3H), 3.74 (s, OCH_3_, 3H), 3.56–3.48 (m, aliphatic, 1H), 2.58–2.48 (m, aliphatic, 1H), 2.21 (dd, aliphatic, *J* = 13.53, 3.98 Hz, 1H), 2.02–1.80 (m, aliphatic, 4H), 1.76–1.56 (m, aliphatic, 2H), 1.52–1.43 (m, aliphatic, 1H); ^13^C NMR (100 MHz, CDCl_3_): 152.3 (C), 152.1 (C), 151.8 (C), 151.6 (C), 142.17 (C), 142.15 (C), 129.1 (C), 128.2 (C), 124.3 (CH), 122.6 (CH), 106.9 (CH), 106.6 (CH), 60.8 (OCH_3_), 60.65 (2 OCH_3_), 60.60 (OCH_3_), 55.97 (OCH_3_), 55.94 (OCH_3_), 43.2 (CH), 42.1 (CH), 30.9 (CH_2_), 30.5 (CH_2_), 29.7 (CH_2_), 23.3 (CH_2_). IR (CH_2_Cl_2_, cm^−1^): 2941, 2872, 2835, 1736, 1601, 1494, 1465, 1435, 1416, 1346, 1273, 1232, 1199, 1164, 1138, 1045, 1019, 973, 941, 906, 798, 752, 737, 689; HRMS (ESI) m/z: [M + H]^+^ Calcd for C_24_H_33_O_6_ 417.2277; Found 417.2268.

#### Bromination of compound *11*

A solution of excess Br_2_ (1.0 mL) was added to a solution of compound **11** (0.1 g, 0.24 mmol) and CH_2_Cl_2_ (20 mL) was added dropwise, over 10 min, with stirring, at RT. After stirring at the same temperature for 20 min, Br_2_ and the solvent in the reaction mixture were removed by rotoevaporation. Chromatography of the crude products on silica gel (40 g) by eluting with CH_2_Cl_2_ gave tetrabromide **12** (80 mg, 46%, colorless liquid) and tribromide **13** (45 mg, 29%, colorless liquid), respectively.

##### 1,2-Dibromo-3-(2-(2,3-dibromo-4,5,6-trimethoxybenzyl)cyclopentyl)-4,5,6-trimethoxybenzene (12)

^1^H NMR (400 MHz, CDCl_3_): 4.04–3.97 (m, aliphatic, 1H), 3.97 (s, OCH_3_, 3H), 3.90 (s, OCH_3_, 3H), 3.87 (s, OCH_3_, 6H), 3.85 (s, OCH_3_, 3H), 3.78 (s, OCH_3_, 3H), 2.79 (t, *J* = 12.19 Hz, aliphatic, 1H), 2.63–2.56 (m, aliphatic, 1H), 2.31–2.21 (m, aliphatic, 2H), 2.01–1.90 (m, aliphatic, 2H), 1.80–1.71 (m, aliphatic, 1H), 1.60–1.45 (m, aliphatic, 2H); ^13^C NMR (100 MHz, CDCl_3_): 153.3 (C), 151.9 (C), 150.6 (C), 150.2 (C), 146.6 (C), 146.4 (C), 133.84 (C), 133.80 (C), 123.6 (C), 121.5 (C), 116.0 (C), 115.82 (C), 61.0 (OCH_3_), 60.91 (OCH_3_), 60.90 (OCH_3_), 60.88 (2 OCH_3_), 60.7 (OCH_3_), 49.4 (CH), 43.0 (CH), 33.1 (CH_2_), 31.0 (CH_2_), 30.4 (CH_2_), 25.6 (CH_2_); IR (CH_2_Cl_2_, cm^−1^): 2937, 2866, 1562, 1460, 1403, 1392, 1352, 1296, 1265, 1237, 1195, 1138, 1101, 1049, 1007, 962, 740, 705; HRMS (APCI) m/z: [M + Na]^+^ Calcd for C_24_H_28_Br_4_NaO_6_ 750.8517; Found 750.8512.

##### 1,2-Dibromo-3-((2-(5-bromo-2,3,4-trimethoxyphenyl)cyclopentyl)methyl)-4,5,6-trimethoxybenzene (13)

^1^H NMR (400 MHz, CDCl_3_): 7.17 (s, aromatic, 1H), 3.92 (s, OCH_3_, 6H), 3.90 (s, OCH_3_, 3H), 3.87 (s, OCH_3_, 3H), 3.85 (s, OCH_3_, 3H), 3.75 (s, OCH_3_, 3H), 3.54 (dd, *J* = 15.66, 8.03 Hz, aliphatic, 1H), 2.63–2.56 (m, aliphatic, 1H), 2.49 (t, *J* = 12.3 Hz, aliphatic, 1H), 2.27 (dd, *J* = 12.57, 3.34 Hz, 1H), 1.97–1.93 (m, aliphatic, 3H), 1.69–1.54 (m, aliphatic, 3H); ^13^C-NMR (100 MHz, CDCl_3_): 152.0 (2C), 150.2 (C), 149.3 (C), 147.2 (C), 146.3 (C), 133.6 (C), 133.2 (C), 126.3 (CH), 121.5 (C), 116.0 (C), 110.7 (C), 61.0 (OCH_3_), 60.94 (OCH_3_), 60.91 (OCH_3_), 60.90 (OCH_3_), 60.86 (OCH_3_), 60.84 (OCH_3_), 42.4 (CH), 42.2 (CH), 32.3 (CH_2_), 29.49 (CH_2_), 29.47 (CH_2_), 23.1 (CH_2_); IR (CH_2_Cl_2_, cm^−1^): 3051, 2937, 2870, 1732, 1564, 1461, 1421, 1402, 1348, 1294, 1266, 1195, 1148, 1097, 1078, 1049, 1008, 961, 929, 866, 820, 792, 739, 705; HRMS (APCI) m/z: [M + H]^+^ Calcd for C_24_H_30_Br_3_O_6_ 652.9572; Found 652.9591.

### Biological assays

The Ellman [25] approach was employed to examine the impact of synthetic chemicals on the ability to inhibit AChE and BChE activity. AChI/BChI and DTNB were used to quantify the AChE/BChE activity. The sources of the enzymes we studied in this article are as follows: Acetylcholinesterase from Electrophorus electricus (electric eel, CAS No.: 9000-81-1), Butyrylcholinesterase from equine serum (CAS No.: 9001–08-5), and α-Glucosidase from *Saccharomyces cerevisiae* (CAS Number: 9001-42-7). For different concentrations of the sample solution, 100 μL of buffer (1 M, Tris/HCl, pH 8.0) were used to dissolve 10 μL of the sample solution. 50 μL were incubated at 25 °C for 10 min after the AChE/BChE (5.32103 EU) solution was added. The incubation was followed by the addition of DTNB (50 μL, 0.5 mM) [26–28]. Lastly, to start the reaction, 50 μL of bChI/aChI were added. The enzymatic hydrolysis of both substrate molecules was obtained by measuring the spectrophotometric production of the 5-thio-2-nitrobenzoate molecule at a wavelength of 412 nm as an impact of the reaction of DTNB molecule with thiocholine molecule. All compounds **4**–**13** were incorporated into the reaction mixture in various dosages to see how they would affect AChE. Then, the activities of AChE/BChE were evaluated [29].

According to Tao et al*.*’s method [30], the activity of synthesized compounds **4**–**13** on α-glucosidase was measured using the substrate p-nitrophenyl-α-D-glucopyranoside (p-NPG). Samples were made by dissolving 20 mg in 20 mL (EtOH:H_2_O). It was initially combined in 5–200 μL of sample with 700 μL of phosphate buffer (0.15 u/mL, pH 7.4) and 20 μL of enzyme solution [31, 32]. After a pre-incubation period of 10 min at 35 °C, 50 μL of p-NPG was added as the reaction began. Additionally, following the pre-incubation, 50 μL of p-NPG in phosphate buffer (5 mM, pH = 7.4) was added, and the incubation was repeated at 35 °C. A curve was fitted to the data to determine the IC_50_ and K_i_ values. The chemical acarbose was used as a positive control. At 405 nm, absorbances were spectrophotometrically quantified [33]. One mole of substrate is hydrolyzed at a rate of one unit of α-glucosidase per minute (pH: 7.4) [34].

### Molecular docking protocol

Molecular docking studies are commonly carried out to predict the binding interaction energies of the identified ligands with the selected proteins. In molecular docking calculations, the most potent active ligands were optimized with the DFT/B3LYP method and 6–31 + G(d,p) basis using the software Gaussian 09W. The 3D crystal structures of the three selected enzymes were downloaded from the Protein Data Bank (PDB) (https://www.rcsb.org/) as follows: 1E66 for AChE [35], 1P0I for BChE [36], and 5ZCC for α-glucosidase [37]. The hetero groups (other ligands, ions, and water) of the proteins were removed from the PDB formats of the protein structures. The in silico docking analyses were performed on AChE, BChE, and α-glucosidase via AutoDock Vina v.1.5.7 [38]. The grid parameters determined were 80 × 70 × 64 Å^3^ x, y, z dimensions, 1.000 Å space, and 5.085, 65.254, 56.371 x, y, z centers for AChE (PDB: 1E66); 68 × 78 × 76 Å^3^ x, y, z dimensions, 0.375 Å space, and 131.841, 116.094, 38.717 x, y, z centers for BChE (PDB: 1P0I); and 66 × 56 × 90 Å^3^ x, y, z dimensions, 0.825 Å space, and 3.195, 48.279, 82.191 x, y, z centers for α-glucosidase (PDB: 5ZCC) as active residues of the target proteins. According to the results for biological activity, the best positions of the selected compounds **9**, **12**, and **13** were visualized and evaluated using BIOVIA Discovery Studio Visualizer v21.1.0.20298 (https://www.3dsbiovia.com/) and polar hydrogens added.

## Results and discussion

### Synthesis

**Scheme 1:**
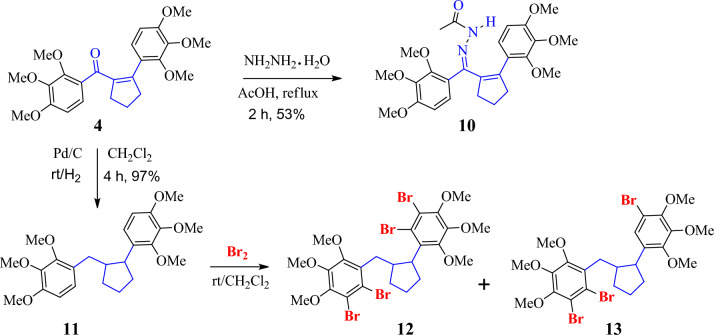
Synthesis of compounds **10–13**

Known compounds **4–9** (Fig. 2) were synthesized from the reaction of 1,2,3-trimethoxybenzene with adipoyl chloride in the presence of AlCl_3_ [7]. Compound **4** is an α,β-unsaturated compound including two benzene rings with trimethoxy groups. Important compounds may be obtained from **4** because it has functional groups such as α,β-unsaturated ketone and benzoyl and phenyl groups with OMe. The compounds to be obtained can be target compounds or intermediates (especially bromides). Refluxing compound **4** with hydrazine monohydride (NH_2_NH_2_.H_2_O) in acetic acid (HOAc) gave a compound that also contained the acetyl group (Scheme 1). This compound should be a hydrazone derivative as **10** because its NMR spectra show an NH hydrogen (at 8.20 ppm as a singlet) and six aliphatic hydrogens (at 2.89–1.95 ppm) in the ^1^H NMR spectrum and sixteen C lines in the double bond region (172.73–106.48 ppm) in the ^13^C NMR spectrum. The reaction of an α,β-unsaturated ketone with hydrazine gives pyrazoline derivatives [3, 21, 39–41]. The inability to observe a pyrazoline derivative instead of a hydrazone derivative in the reaction of compound **4** with hydrazine monohydride may be due to the OMe in the o-position of the phenyl ring attached to the β carbon atom of **4**. The structure of hydrazone **10** can be either *trans-* or *cis*-structure. As shown Scheme 1, this structure was assumed to be in a trans structure, which would be more stable when steric effects are taken into account.

A catalytic hydrogenation reaction (H_2_, Pd/C) of compound **4** was performed, and one product was isolated from this reaction. It is known that in the reduction of α,β-unsaturated ketones such as **4**, first double bound group, and then the carbonyl group are reduced [3]. It is very difficult to determine the configurations of the vicinal bulky (substituted benzyl and phenyl) groups in the quintuple ring in this product by NMR spectroscopy. Having obtained a single crystal of this product, its structure is easy to explain by X-ray analysis. However, we were unable to obtain its crystal. Double bonds are reduced in the *cis*-configuration by catalytic hydrogenation. A *trans*-isomer was obtained in the reduction of the cyclohexene derivative compound by catalytic hydrogenation [42]. In the catalytic hydrogenation reaction of **4**, the reduction product was assumed to be **11** due to the highly steric effect.

The reaction of compound **11** with molecular bromine performed, and two products were obtained from the chromatography of crude products on silica gel. When the ^1^H NMR spectra of these two products were examined, it was observed that one of them had no aromatic hydrogen peak while the other one had an aromatic hydrogen peak (at 7.17 ppm as s). The product without one aromatic hydrogen should be tetrabromide **12** and the other a tribromide compound. It was accepted that the structure of the tribromide was **13** and the steric state was effective in its formation. ^1^H NMR spectrum of the tetrabromide **12** is consistent with the proposed structure. However, we had a doubt as to whether the peak at 49.4 ppm in the ^13^C NMR spectrum belongs to the compound **12**. To clarify this, the HMQC spectrum of the compound was taken. Due to the slow rotation, the small and broad-looking peak should be the benzylic CH adjacent to the phenyl ring. The HMQC spectrum of the compound **12** is given in the Supporting Information file.

### Biological activity

In the present study, preparated compounds were evaluated against some metabolic enzymes (α-Glu, AChE, and BChE) (Table 1).

1. Long-chain dietary carbohydrates are hydrolyzed by the enzyme α-glucosidase in the small intestine to form monosaccharide units that enter the bloodstream and cause hyperglycemia [43]. As a result, α-glucosidase inhibition has become a crucial therapeutic target with the potential to lower blood sugar levels by slowing down the digestion of carbohydrates [44]. The α-glucosidase inhibitors (Fig. 3), however, primarily influence hyperglycemia without directly influencing insulin secretion [45]. The data obtained demonstrated that some of the preparated compounds with K_i_ values ranging from 25.47 ± 4.46 to 48.87 ± 7.33 nM exhibited inhibitory activity more than the positive control acarbose, with a K_i_ value of 43.06 ± 6.07 nM for α-glucosidase Among the compounds synthesized, the most potent were **9**, **12**, and **6**, with K_i_ values of 25.47 ± 4.46, 27.42 ± 5.17, and 29.13 ± 4.25 nM, respectively. These compounds were 1- to 1.5-fold more potent than the positive control acarbose. On the other hand, compounds **13**, **11**, and **8** showed average and near inhibition potentials, with K_i_ values of 35.63 ± 3.95, 38.14 ± 5.48, and 38.51 ± 4.25 nM, respectively, against this enzyme. Additionally, the K_i_ results for compounds **5**, **7**, and **4** (44.71 ± 6.61, 46.47 ± 6.34, and 48.87 ± 7.33 nM, respectively) were similar to those for the standard (K_i_: 43.06 ± 6.07 nM). As a result, they are utilized as monotherapy in the treatment of moderate diabetes and are regarded as the first-line oral sugar-reducing medications. In cases of acute diabetes, they are utilized in combination with insulin or other drugs [46]. However, research has found that these drugs also have a number of side effects, including diarrhea, abdominal pain, bloating, and flatulence. These factors have led researchers to concentrate on discovering novel substances and, in recent years, a large number of novel substances have been identified as α-glucosidase inhibitors [47]. The small intestine's enzyme α-glucosidase has the ability to hydrolyze the α − 1 $\dot{ \to }$ 4 bond of oligosaccharides or disaccharides, resulting in the production of glucose. Evidently, the conversion of carbohydrates into blood glucose might be significantly inhibited by inhibiting the activity of α-glucosidase. As a result, the reduced adsorption of glucose could lower the postprandial blood glucose level, which is a useful way to lessen postprandial hyperglycemia symptoms in T2DM patients [40, 45]. Indeed, α-glucosidase inhibitors can be candidates for anti-diabetic drugs in living cells; therefore, we detected the compounds in the present study as new inhibitors in vitro (Fig. 2). We intend to study these inhibitors in vivo in our future studies.

**Fig. 3:**
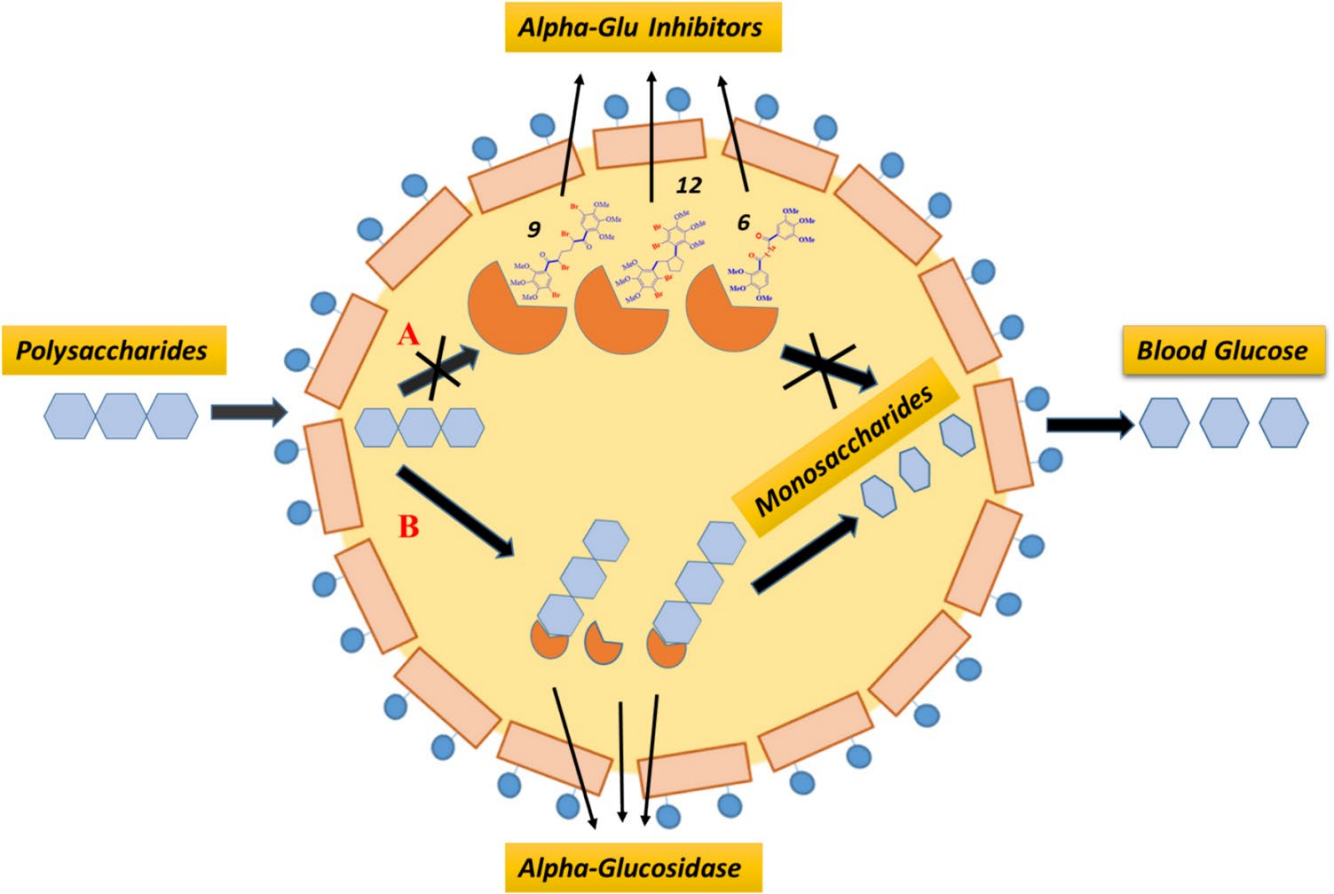
Mechanism of α-glucosidase inhibitors
2. The AChE inhibitors donepezil and galantamine, as well as a dual inhibitor called rivastigmine, are currently the only anticholinesterase medications that the FDA has approved. They have a positive impact on the cognitive, functional, and behavioral signs and symptoms of the condition [48]. Although these medications are generally beneficial, several side effects have been noted, including exhaustion, nausea, vomiting, muscle cramps, diarrhea, and loss of appetite. In this regard, much effort has been made to create novel dual cholinesterase inhibitors for the treatment of AD’s neurological problems. Finding new candidate medications that target various AD-related enzymes by combining in vitro and in silico research represents a valuable strategy [49, 50]. The K_i_ values of the test compounds demonstrated that the most active compounds against AChE were **9**, **12**, and** 5** (K_i_: 45.53 ± 7.35, 61.15 ± 5.26, and 87.91 ± 7.27 nM, respectively) (Table 1). Moreover, for AChE, the IC_50_ values of tacrine (TAC) as the positive control and the novel compounds examined in the present study were in the following order: **9** (49.84 nM, r^2^: 0.9391) < **12** (65.52 nM, r^2^: 0.9850) < **5** (92.25 nM, r^2^: 0.9550) < **4** (102.32 nM, r^2^: 0.9891) < **13** (124.16 nM, r^2^: 0.9943) < **TAC** (241.61 nM, r^2^: 0.9366). The most potent compounds among the synthesized derivatives against BChE were **12**, **10**, and **11** with K_i_ values of 84.30 ± 9.92, 131.22 ± 14.54, and 144.63 ± 15.46 nM, respectively. The data obtained demonstrated that the synthesized compounds, with K_i_ values ranging from 84.30 ± 9.92 to 622.10 ± 35.14 nM, exhibited greater inhibitory activity than the positive control tacrine, with a K_i_ value of 352.15 ± 23.55 nM, BChE except for compounds **4**, **7**, and **9** (Table 1). Additionally, for AChE, the *Ki* values of tacrine (TAC) as the positive control and some compounds examined in the present study were in the following order: **9** (45.53 ± 7.35 nM) < **12** (61.15 ± 5.26 nM) < **5** (87.91 ± 7.27 nM) < **4** (99.72 ± 11.44 nM) < **13** (108.14 ± 5.14 nM) < **TAC** (226.52 ± 19.43 nM). Moreover, for BChE, the Ki values of tacrine (TAC) as the positive control and some compounds examined in the present study were in the following order: **12** (84.30 ± 9.92 nM) < **10** (131.22 ± 14.54 nM) < **11** (144.63 ± 15.46 nM) < **5** (175.62 ± 23.80 nM) < **13** (205.13 ± 17.41 nM) < **TAC** (352.15 ± 23.55 nM). Also, for α-Glu, the *Ki* values of ACR as the positive control and some compounds examined in the present study were in the following order: **9** (25.47 ± 4.46 nM) < **12** (27.42 ± 5.17 nM) < **6** (29.13 ± 4.25 nM) < **10** (31.23 ± 5.54 nM) < **13** (35.63 ± 3.95 nM) < **ACR** (43.06 ± 6.07 nM).

**Table 1:** The enzyme inhibition results of the examined compounds against AChE, BChE, and α-glucosidase (α-Glu)

Compounds	IC_50_ (nM)						K_i_ (nM)		
	AChE	r^2^	BChE	r^2^	α-Glu	r^2^	AChE	BChE	α-Glu
**4**	102.32	0.9891	414.35	0.9141	41.57	0.9463	99.72 ± 11.44	402.73 ± 32.31	48.87 ± 7.33
**5**	92.25	0.9550	187.16	0.9370	39.65	0.9836	87.91 ± 7.27	175.62 ± 23.80	44.71 ± 6.61
**6**	189.45	0.9354	225.49	0.9665	27.23	0.9821	176.52 ± 22.61	212.42 ± 13.71	29.13 ± 4.25
**7**	643.65	0.9865	646.46	0.9476	43.20	0.9887	631.96 ± 18.88	622.10 ± 35.14	46.47 ± 6.34
**8**	173.73	0.9268	221.55	0.9782	36.36	0.9951	171.23 ± 14.13	209.21 ± 14.66	38.51 ± 4.25
**9**	49.84	0.9391	450.27	0.9841	21.12	0.9770	45.53 ± 7.35	428.42 ± 27.42	25.47 ± 4.46
**10**	205.18	0.9302	135.27	0.9752	28.42	0.9969	196.28 ± 13.73	131.22 ± 14.54	31.23 ± 5.54
**11**	169.53	0.9303	155.45	0.9890	33.38	0.9830	151.46 ± 13.34	144.63 ± 15.46	38.14 ± 5.48
**12**	65.52	0.9850	92.74	0.9526	23.91	0.9977	61.15 ± 5.26	84.30 ± 9.92	27.42 ± 5.17
**13**	124.16	0.9943	217.40	0.9239	31.63	0.9870	108.14 ± 5.14	205.13 ± 17.41	35.63 ± 3.95
TAC^a^	241.61	0.9366	374.62	0.9394	–	–	226.52 ± 19.43	352.15 ± 23.55	–
ACR^b^	–	–	–	–	40.07	–	–	–	43.06 ± 6.07

^a^Tacrine** (**TAC) was used as a control for AChE and BChE

^b^Acarbose (ACR) was used as a control for α-glucosidase

### Molecular docking studies

Molecular docking simulations were performed to predict possible interactions between the most active compounds among the preparated compounds **4**–**13** and the active sites of the three enzymes (AChE, BChE, and α-glucosidase). In this study, in vitro and docking studies were also carried out by adding compounds **4–9**, including the derivatives of these compounds, in addition to the new compounds **10–13**. According to the results of the in vitro analysis, tetrabromide **9** showed a stronger inhibitory activity for both AChE and α-glucosidase target enzymes than the control molecule, tacrine (Table 1). On the other hand, tetrabromide **12** showed the strongest activity for the BChE target enzyme, while it showed the second strongest inhibitory activity for both AChE and α-glucosidase. What is surprising here is that tribromide **13**, which is in the structure of tetrabromide **12** and has only one less bromine atom, has much less activity than tetrabromides **9** and **12** with respect to K_i_ values (Table 1). Therefore, the interactions between **9**, **12**, and **13** with α-glucosidase (PDB: 5ZCC) [37] were simulated to examine the effect of bromine atom on the inhibition mechanism. Moreover, in silico molecular docking analyses of tetrabromide **9** with AChE (PDB: 1E66) [35] and of tetrabromide **12** with BChE (PDB: 1P0I) [36] receptors were conducted.

**Fig. 4:**
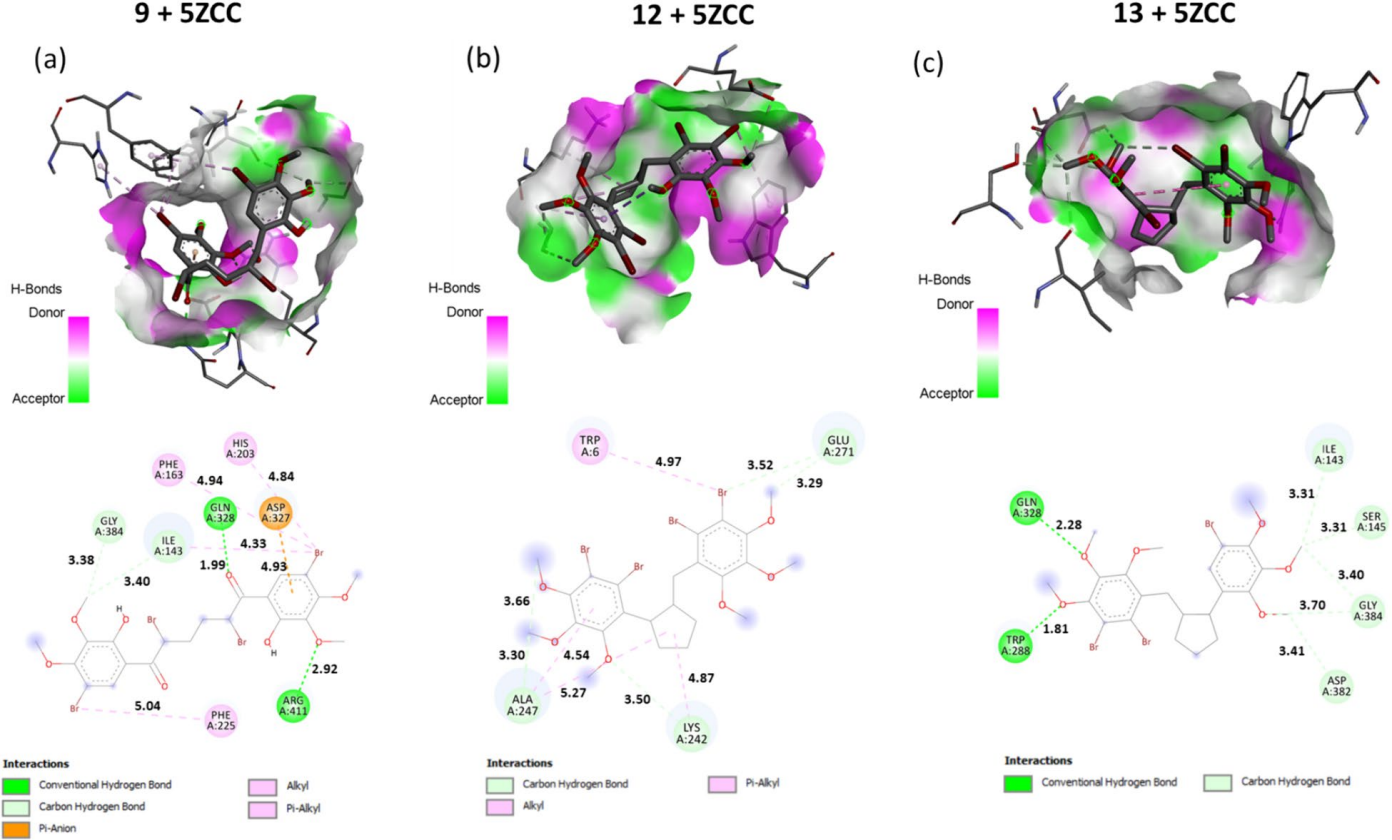
3D and 2D Molecular docking results of tetrabromide **9** (**a**), tetrabromide **12** (**b**), and tribromide **13** (**c**) with α-glucosidase (PDB: 5ZCC)

The three-dimensional (3D) and two-dimensional (2D) graphs obtained for docked **9**, **12**, and **13** into the active site of α-glucosidase by considering the best bonding results are shown in Fig. 4. For **9**, **12**, and **13** with α-glucosidase (PDB: 5ZCC), the molecular docking scores (the best binding energy values) calculated were − 6.5, − 6.4, and − 6.1 kcal/mol, respectively. As can be clearly seen in Fig. 4, the higher activity of **9** compared to **12** and **13** mainly refers to the number of both hydrogen bonds and other interactions formed in the **9**-α-glucosidase complex. Two conventional hydrogen bonds and two carbon hydrogen bonds are formed between **9** and amino acid residues of α-glucosidase active sites Gln328, Arg411 and Gly384, Ile143, respectively. Compound **9** forms one strong hydrogen bond between the oxygen atom of the carbonyl group and Gln328 of length 1.99 Å. The second hydrogen bond is formed between the oxygen atom of the methoxy group and Arg411 of length 2.92 Å. The other five non-hydrogen bonding interactions are of type π-anion, alkyl, and π-alkyl formed with the amino acid residues Asp327, His203, Phe163, Ile143, and Phe225.

In compound **12**, the five carbon hydrogen bonds occurred between Glu271 and C36 of length 3.29 Å, between Glu271 and Br60 of length 3.52 Å, between Ala247 and C48 of length 3.66 Å, between Ala247 and C44 of length 3.30 Å, and between Lys242 and O25 of length 350 Å. The other interactions, which are alkyl and π-alkyl type interactions were observed as shown in Fig. 4. These four non-hydrogen bonding interactions are of alkyl and π-alkyl types, formed with the amino acid residues Trp6, Lys242, and Ala247 (Fig. 4).

**Fig. 5:**
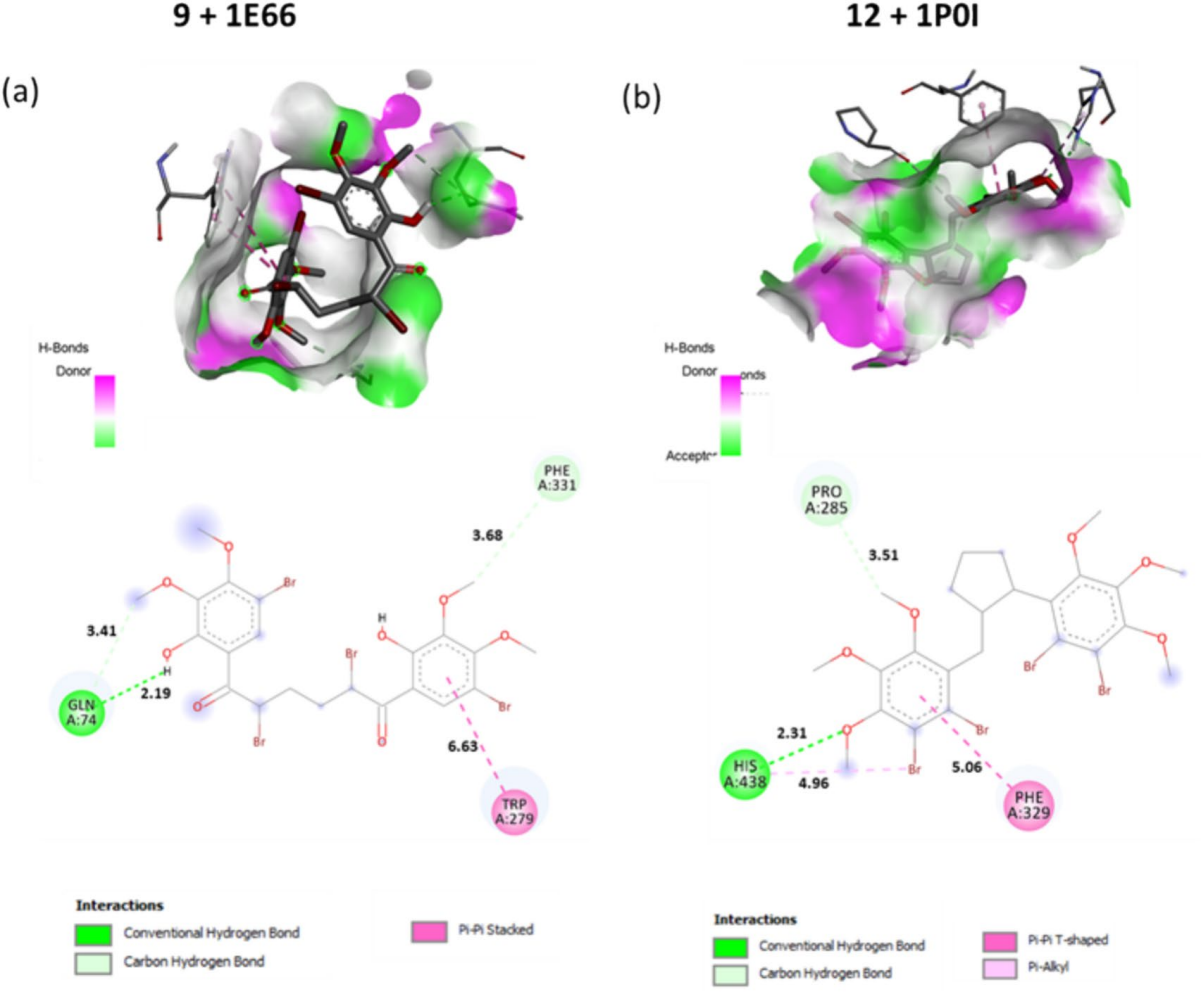
3D and 2D Molecular docking results: **a** compound **9** with AChE (PDB: 1E66), **b** compound **12** with BChE (PDB: 1P0I)

Furthermore, in compound **13**, one of the two hydrogen bonds, of length 1.81 Å, is formed between the oxygen atom of the methoxy group and the amino acid residue Trp288, while the other, of length 2.28 Å, is formed between the oxygen atom of the methoxy group and the amino acid residue Gln328. The five carbon hydrogen bonding interactions are also formed between Ile143 and C44 of length 3.31 Å, between Ser145 and C44 of length 3.31 Å, between Gly384 and C44 of length 3.40 Å, between Asp382 and C40 of length 3.41 Å, and between Gly384 and O25 of length 3.70 Å (Fig. 5). As a result, as seen in Fig. 4, in this section where we examined the effect of the bromine atom on the binding, while there were four and two interactions between the bromine atoms in tetrabromides **9** and **12** and the amino acid residues of α-glucosidase, respectively, no interaction was observed with the bromine atoms in tribromide **13**. While compounds **9** and **12**, which contain four bromine atoms, show a very strong activity against α-glucosidase, it is interesting that compound **13** which has only one atom missing shows a weak activity despite having the same skeletal structure as compound **12**. This shows that the number of bromine atoms in the molecule has an effect on the inhibition activity.

For compound **9**, showing the strongest activity against AChE (PDB: 1E66), the best binding energy value calculated was − 6.5 kcal/mol. Compound **9** forms one strong hydrogen bond between the hydrogen (H30) of the hydroxyl group and the amino acid residue Gln74 of length 2.19 Å. The other interactions of **9** involving two carbon hydrogen bonds and π-π stacked interaction were formed with the amino acid residues Gln74, Phe331, and Trp279 (Fig. 5).

For compound **12**, showing the strongest activity against BChE (PDB: 1P0I), the molecular docking score calculated was − 7.4 kcal/mol. Compound **12** forms a strong hydrogen bond between the oxygen atom (O24) of the methoxy group and His438 of length 2.31 Å. The other interactions of **12** involving one carbon hydrogen bond, π-π T-shaped, and π-alkyl were formed between Pro285 and C28 with 3.51 Å, between Phe329 and the phenyl ring of the benzyl group with 5.06 Å, and between His438 and Br60 with 4.96 Å, respectively (Fig. 5).

### DFT studies

#### Molecular structure analysis

All computations were performed using the software Gaussian 09W [51]. Geometric optimizations of the compounds were performed using density functional theory method at the B3LYP with 6–31 + G(d,p) basis set [52, 53]. The results were visualized by the visualization software CYLview [54] and GaussView 5.0 [55]. DFT is an excellent method often used to describe molecular structure and stability, mechanistic insights, and molecular interactions [56–61].

In our previous study, DFT studies including structural properties, natural bond orbital analysis of donor–acceptor interactions, charges on the atoms, comparison of intramolecular hydrogen bonds, and quantum chemical reactivity identifiers for compounds **4**–**9** were performed using two functional levels, B3LYP and M06-2X, and examined in detail [7]. Moreover, the experimental ^1^H and ^13^C NMR chemical shifts were compared with the calculated values.

**Fig. 6:**
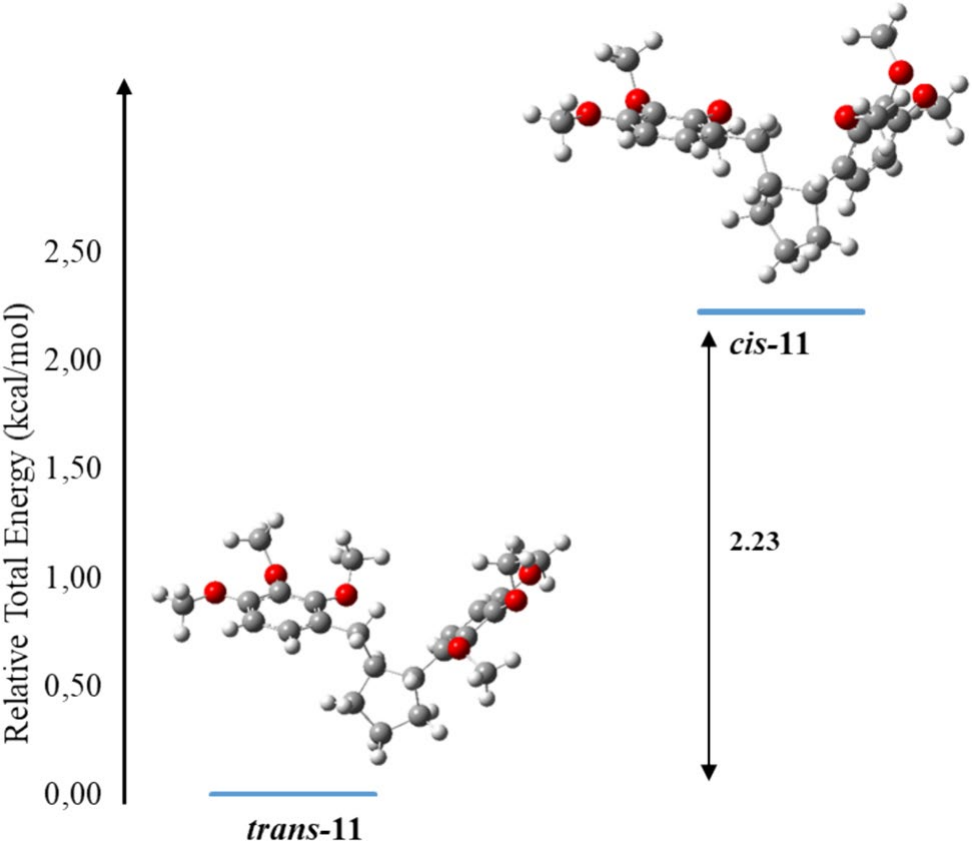
Relative total energy profiles of *cis***-** and *trans***-11**

**Fig. 7:**
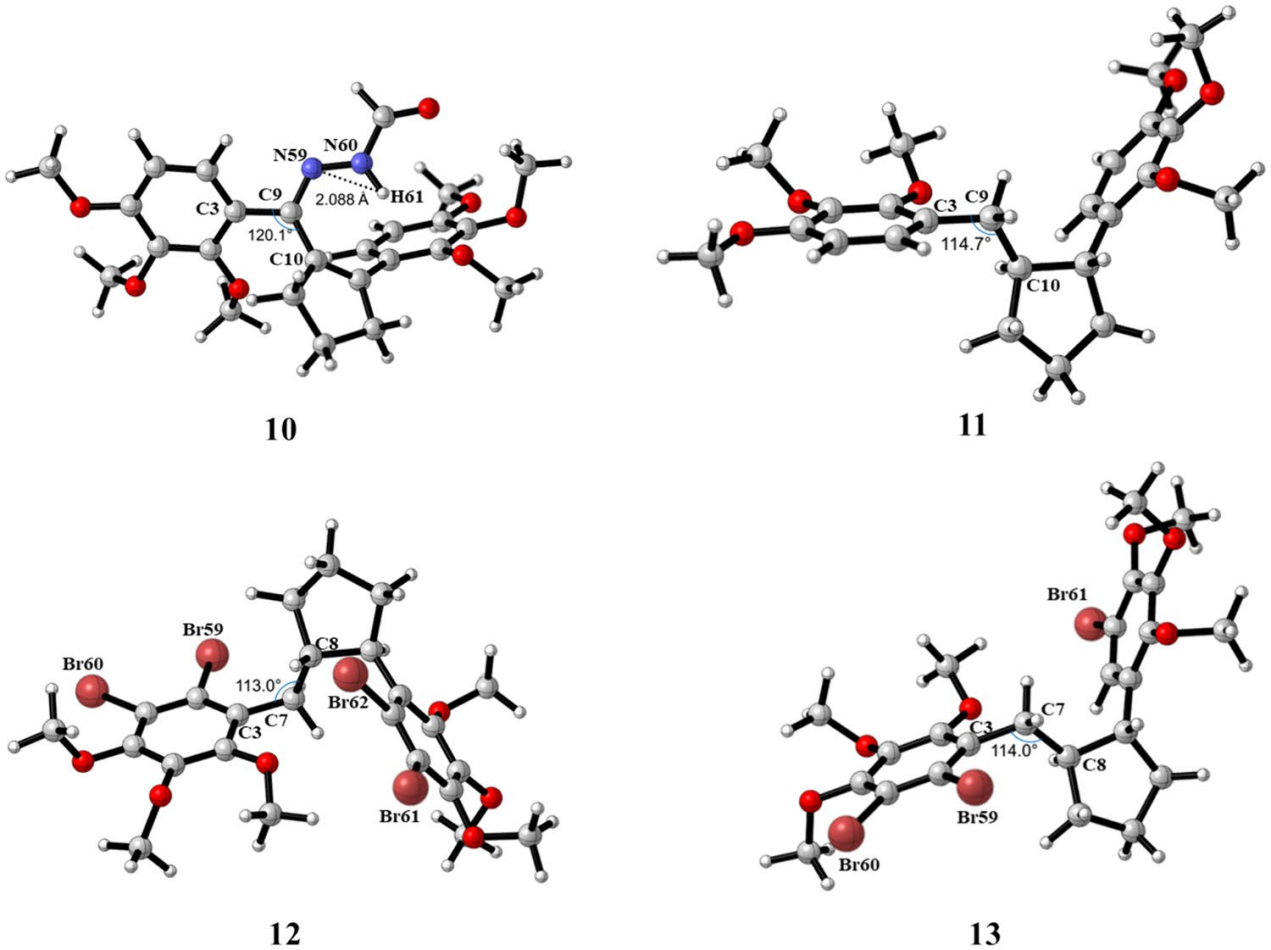
Optimized geometries of **10–13** using the B3LYP/6–31 + G(d,p) basis set

In the synthesis section, the molecular structure and total energies of compound **4** were examined in order to support that the product formed in the catalytic hydrogenation could be a trans isomer. To compare the stabilities of *cis***-11** and *trans***-11**, both compounds were optimized using the B3LYP/6–31 + G(d,p) basis set (Fig. 6). The relative total energy for *trans***-11** carried out at the B3LYP/6–31 + G(d,p) level is lower for 2.23 kcal/mol than the energy for ***cis*-11** (Fig. 6). The theoretical calculations support the proposed configuration of the groups in the cyclopentane ring. Compounds **10**–**13** were optimized at DFT/B3LYP/6–31 + G(d,p) level of theory in the gas phase (Fig. 7). Some selected structural parameters of compounds **10**–**13** are given in Table S1. The dihedral angles C4–C3–C9–C10 and C19–C18–C12–C10 for **10** and **11** are − 41.6° and − 76.0°, and − 52.2° and − 55.5°, respectively. According to these values, the phenyl and cyclopentene rings for compound **10** were deviated from the plane due to weak conjugation.

#### Natural bond orbital (NBO) analysis

NBO analysis is a useful computational approach used both to investigate intramolecular interactions, hydrogen bonds, and charge transfers and to calculate hyperconjugative interactions between atoms and molecules. A larger stabilization energy (E^(2)^) value indicates that there is more intense interaction between electron donors and electron acceptors, and the extend of the conjugation of the whole system is also greater. E^(2)^ values can be calculated by second order perturbation theory using the following equation [62, 63]:

$$ E^{\left( 2 \right)} = \Delta E_{ij} = q_i \frac{(Fij)^2 }{{\varepsilon_j - \varepsilon_i }} $$

In this equation, q_i_ is “the donor orbital occupancy”, *ɛ*_*j*_ and *ɛ*_*i*_ are diagonal elements, and (*Fij*) is “the off-diagonal NBO Fock matrix element”.

Selected NBO donor–acceptor interactions in the analysis results of compounds **10**–**13** are given in Table 2. The significant hyperconjugative interactions and the stabilization energies were observed by NBO analysis. In compound **10**, strong intramolecular hydrogen bonding was observed from LP(1) N59 to antibonding orbitals σ*(N60-H61) with a stabilization energy of 9.35 kcal/mol. Moreover, considerably high hyperconjugative interaction values were observed in compounds **12** and **13** due to the bromine atoms attached to the aromatic ring. The hyperconjugative interaction energies of LP(2) O22 → σ*(C19–C21), LP(2) O25 → σ*(C19–C21), LP(1) Br62 → σ*(C10–H58) and LP(2) Br62 → σ*(C19–C21) for **12** are 1035.83, 1049.99, 3590.65 and 3621.41 kcal/mol, respectively. The hyperconjugative interaction energies of LP(2) O27 → σ*(C48-H51), LP(1) Br60 → σ*(C44-H46) and LP(1) Br60 → σ*(C48-H51) for **13** are 1266.37, 233.74 and 228.85 kcal/mol, respectively.

**Table 2:** Selected NBO donor–acceptor interactions for the intramolecular interactions of **10–13** using B3LYP/6–31 + G(d,p) basis set

Donor NBO (i)	Acceptor NBO (j)	E^(2)^^a^ (kcal/mol)	E(j) − E(i)^b^ (a.u)	F(i,j)^c^ (a.u)
**10**				
σ (C1–C6)	σ* (C5–C6)	3.20	1.25	0.057
σ (C20–C23)	σ* (C18–C20)	3.22	1.27	0.057
π (C1–C2)	π* (C5–C6)	24.17	0.26	0.074
π (C3–C4)	π* (C1–C2)	23.57	0.28	0.073
LP(2) O27	σ* (C26–H38)	6.31	0.76	0.063
LP(2) O31	π* (C21–C24)	29.88	0.34	0.096
LP(1) N59	σ* (N60–H61)	9.35	0.80	0.078
LP(1) N60	π* (C9–N59)	29.58	0.28	0.085
**11**				
σ (C1–C6)	σ* (C5–C6)	3.28	1.25	0.057
σ (C21–C24)	σ* (C23–C24)	3.21	1.26	0.057
π (C2–C3)	π* (C4–C5)	21.08	0.27	0.069
π (C18–C19)	π* (C20–C23)	22.08	0.27	0.070
LP(2) O27	σ* (C36–H38)	6.38	0.76	0.063
LP(2) O29	σ* (C44–H45)	6.43	0.78	0.064
LP(2) O31	π* (C21–C24)	28.61	0.34	0.095
**12**				
σ (C3–C4)	σ* (C10–H58)	424.84	0.57	0.441
σ (C18–O25)	σ* (C10–H58)	447.40	1.26	0.672
σ (C18–O25)	σ* (C19–C21)	661.66	1.34	0.846
LP(2) O22	σ* (C10–H58)	376.83	0.44	0.376
LP(2) O22	σ* (C19–C21)	1035.83	0.52	0.673
LP(2) O25	σ* (C19–C21)	1049.99	0.68	0.760
LP(1) Br62	σ* (C10–H58)	254.52	0.33	0.259
LP(1) Br62	σ* (C19–C21)	429.57	0.41	0.377
LP(1) Br62	σ* (C10–H58)	3590.65	0.03	0.274
LP(2) Br62	σ* (C19–C21)	3621.41	0.11	0.552
**13**				
σ (C1–Br60)	σ* (C40–H42)	150.92	0.58	0.263
σ (C1–Br60)	σ* (C44–H56)	193.32	0.62	0.308
σ (C16–C17)	σ* (C48–H51)	558.96	2.34	1.027
LP(2) O25	σ* (C40–H42)	58.92	0.18	0.094
LP(1) O27	σ* (C44–H46)	358.78	0.57	0.406
LP(2) O27	σ* (C48–H51)	1266.37	2.99	1.762
LP(1) Br60	σ* (C44–H46)	233.74	1.04	0.441
LP(1) Br60	σ* (C48–H51)	228.85	2.43	0.668

^a^ E^(2)^ means energy of hyper conjugative interaction (stabilization energy)

^b^ Energy difference between donor and acceptor i and j NBO orbital

^c^ F(i, j) is the Fork matrix element between i and j NBO orbital

In addition, the NBO analysis also shows the natural charge on the atoms in the molecule. Natural charges and Mulliken atomic charges are listed in Tables S2 and S3. The charge distributions on the atoms give important information about how the charge transfers in the molecule will take place. In general, natural population analysis data for atomic charges are more preferable than Mulliken charges. In compound **10**, the charge of the carbon atom (C16) with the largest negative charge is − 0.47822, while in compound **11**, the charge of the carbon atom (C13) is − 0.48081. For **10**, it was observed that N60 (− 0.46622) is a more electronegative atom than N59 (− 0.25729). The oxygen (O63) of the carbonyl group draws the electrons from the nitrogen atom (N60), and therefore N60 atom has a larger negative charge. The carbon atoms C4, C5, C6, C18, C20 and C21 have a positive charge in the charge allocation of compound **12** and **13**, indicating that they are acceptor atoms. In both compounds, the other carbon atoms have a negative charge, and they operate as donors. In compounds **10**, **11**, **12** and **13**, the highest positive charge for all hydrogen atoms was found as H61 (0.42659), H59 (0.26498), H58 (0.27231) and H55 (0.27231), respectively.

#### Frontier molecular orbital (FMO) analysis

**Fig. 8:**
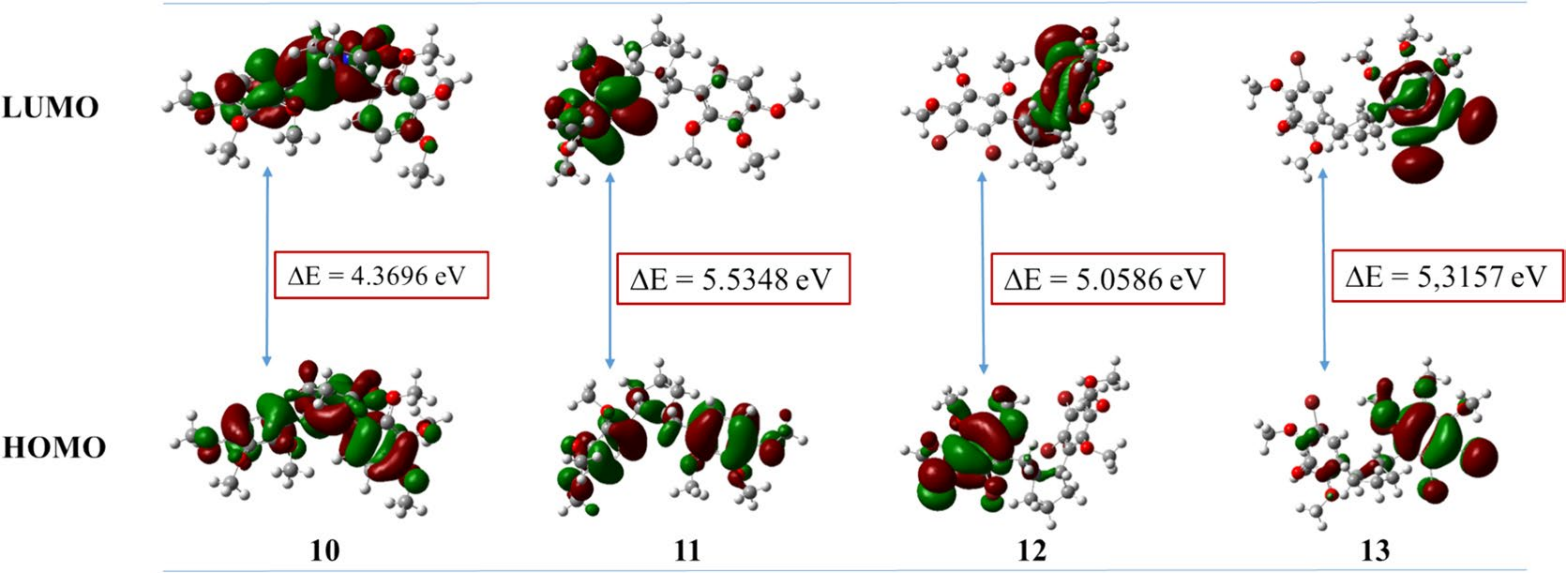
The frontier molecular orbitals of 10–13 using the B3LYP/6–31 + G(d,p) basis set

The energy of the frontier molecular orbitals (HOMO and LUMO) is often used to make inferences about the chemical reactivity and kinetic stability of the molecules of interest. The HOMO–LUMO gap helps determine the chemical reactivity of the molecule. According to this approach, a molecule with a small boundary orbital gap becomes more polarized, facilitating the transition of electrons from HOMO to LUMO, thus having low kinetic stability and high chemical reactivity [64–66]. The HOMO and LUMO energies for compounds **10**–**13** were calculated using the DFT/B3LYP/6–31 + G(d,p) basis set. The charge density distributions of the HOMO and LUMO orbitals for compounds **10**–**13** are shown in Fig. 8.

It is clearly seen in Fig. 8 that, in the case of the HOMO, the charges density for compounds **10** and **11** is completely localized on almost all the molecules. In the case of the LUMO, the charge density for compound **11** is concentrated on the phenyl ring attached to the cyclopentyl ring, while the charge for compound **10** is more densely distributed on the other phenyl ring and the acetohydrazone group attached to it and the cyclopentyl ring.

Among compounds **4–13**, based on the K_i_ values obtained, compound **9**, having four bromine atoms, had the most powerful inhibition against AChE and α-glucosidase, with the K_i_ values of 45.53 nM and 25.47 nM, respectively. It was also observed that compound **12**, the other compound with four bromine atoms, was the second most active compound against AChE and α-glucosidase. Moreover, compound **12** showed the strongest activity against BChE, with a K_i_ value of 84.30 nM. Compound **10**, including an acetohydrazone group, was the second most active compound against BChE, with a K_i_ value of 131.22 nM. What is surprising here is that compound **13**, which has three bromine atoms in similar positions, showed much less activity than compounds **9** and **12** for AChE, BChE, and α-glucosidase, with K_i_ values of 108.14, 205.13, and 35.63 nM, respectively.

**Fig. 9:**
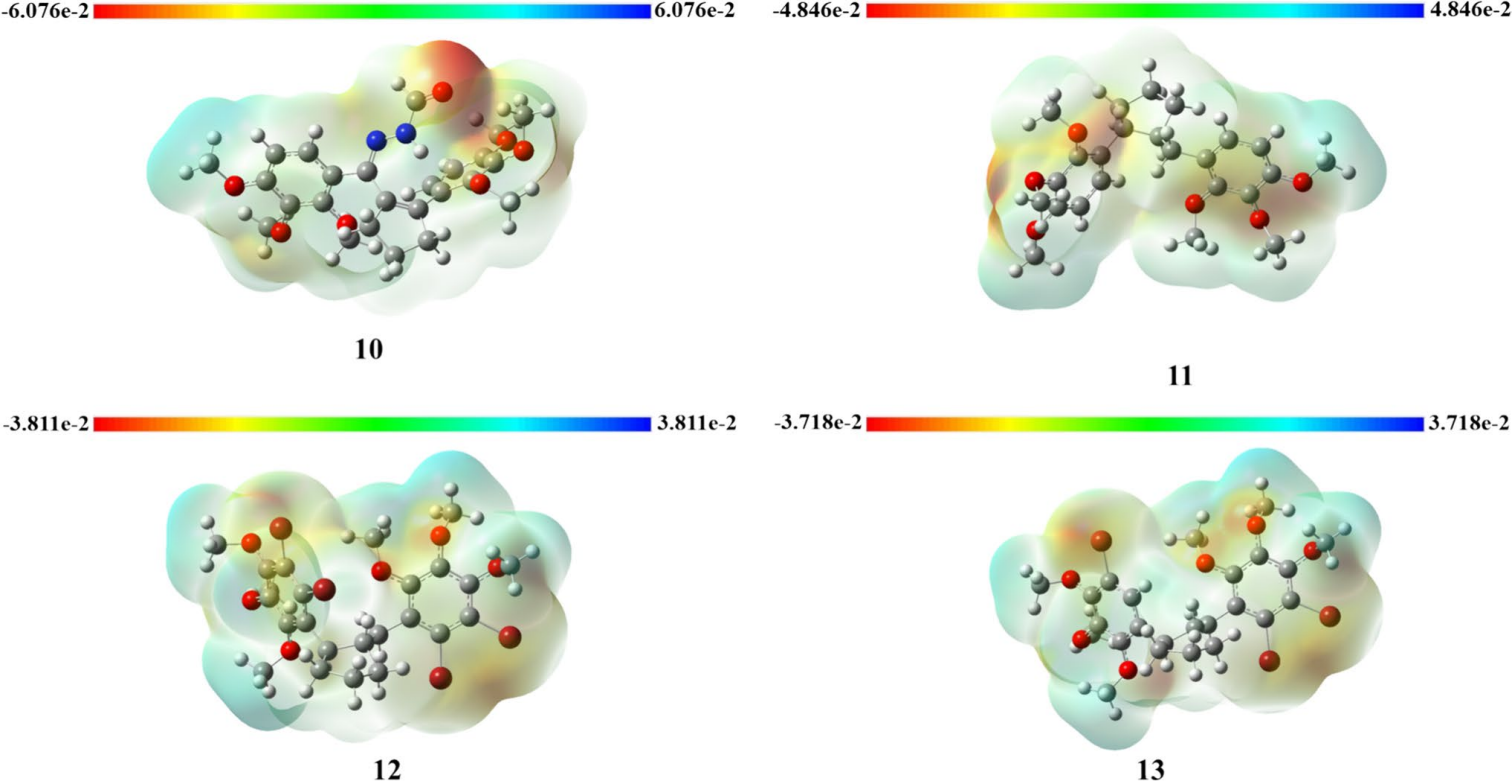
The MEP surfaces of 10–13 using the B3LYP/6–31 + G(d,p) basis set

To explain this situation, we examined frontier molecular orbital images (Fig. 8) and MEP surfaces (Fig. 9). The frontier molecular orbital analysis of compound **9** was performed in detail in our previous study [7]. In the case of the HOMO, the charge densities for the symmetric compound **9** spread out from the aromatic ring to the carbonyl group. In the case of the LUMO, the charges spread from the phenyl ring to the carbon atom to which the bromine atom is bonded. In the case of the HOMO, the charges for compound **12** are located on the benzyl group, while in the case of the LUMO, the charges are located on the phenyl group attached to the cyclopentene ring. In the case of the HOMO, the charges for compound **13** are mostly located on the phenyl ring containing two bromine atoms, with a very small charge on the other phenyl ring. In the case of the LUMO, the charges are spread only on the phenyl ring containing dibromine atoms.

The quantum chemical reactivity parameters are commonly used to make inferences about the chemical behavior of molecules. These parameters for **10–13** are given in Table 3. According to Koopmans’ theorem [67], the HOMO and LUMO energies are related to electron affinity (*A* = − LUMO) and ionization energy (*I* = − HOMO). Several global reactivity identifiers such as electron affinity (*A*), ionization potential (*I*), global chemical hardness (η), global softness (σ), electronegativity (χ), electrophilicity (*ω*), and chemical potential (*μ*) have been calculated using by the following equations [68–70].

$$ \eta = \left( {I-A} \right)/{2} $$

$$ \sigma = {1}/\eta $$

$$ \chi = \left( {I + A} \right)/2 $$

$$ \mu = -\left( {I + A} \right)/{2} $$

$$ \omega = \mu^{2} /{2}\eta $$

**Table 3:** The quantum chemical reactivity parameters (eV) of **10–13**

	HOMO	LUMO	ΔE	*I*	*A*	*ƞ*	*σ*	*χ*	*μ*	*ω*
**10**	− 5.6532	− 1.2836	4.3696	5.6532	1.2836	2.1848	0.4577	3.4684	− 3.4684	2.7530
**11**	− 5.7098	− 0.1750	5.5348	5.7098	0.1750	2.7674	0.3613	2.9424	− 2.9424	1.5642
**12**	− 6.1299	− 1.0713	5.0586	6.1299	1.0713	2.5293	0.3954	3.6006	− 3.6006	2.5628
**13**	− 6.2592	− 0.9434	5.3157	6.2592	0.9434	2.6579	0.3762	3.6013	− 3.6013	2.4398

#### Molecular electrostatic potential (MEP) analysis

The MEP surfaces of compounds **10–13** are illustrated in Fig. 9. MEP analysis provides visual information about determining reactive sites for electrophilic and nucleophilic attacks and three-dimensional plots of total charge density of the molecule [71]. In Fig. 9, red shows the negative side of the molecule, consisting of electron-rich regions, while blue depicts the positive side, consisting of electron-poor regions. The electron-rich red regions in **10** are mainly located around the carbonyl group of the acetohydrazide group. For compounds **10**–**13**, the electron-rich (red) regions are mainly located around the oxygens of the methoxy groups and the bromine atoms.

## Conclusions

The products **10** and **11** were obtained from the reactions of known **4** with hydrazine hydrate and catalytic hydrogenation, respectively. Bromination of **11** were performed, and tetrabromide **12** and tribromide **13** were obtained from the reaction. It should be that configurations of molecules are preserved in the products (except for **11**) of reactants containing cyclopentene or cyclopentane units. The reaction products were purified and their structures interpreted. When the biological activities of the prepared compounds **4–13** are examined. Most of the prepared compounds inhibited AChE and BChE better than the positive control tacrine and α-glucosidase better than acarbose. It is a very interesting finding that the activities of the tetrabromides **9** and **12**, in which each has two bromines attached to each phenyl ring, have much stronger activity than the tribrominated derivatives against AChE, BChE, and α-glucosidase. In silico molecular docking studies were also conducted on compounds **9**, **12**, and **13** to predict their binding modes with the active sites of the respective enzymes. Because of the interactions between the bromine atoms of tetrabromides **9** and **12**, and amino acid residues of the α-glucosidase, as well as the absence of any interaction with the bromine atoms in tribromide **13**, these important findings observed will also shed light on the binding mechanism of ligands to the receptor. DFT studies for compounds **10**–**13** were carried out for a better understanding of the potential AChE, BChE, and α-glucosidase activity mechanism.

## Supplementary Information

Below is the link to the electronic supplementary material.

Supplementary file1 (DOCX 27102 KB)

## Data Availability

All data are available in the article and its supplementary material.
