# Supplementary material for: Investigation of cholinesterase and α-glucosidase enzyme activities, and molecular docking and dft studies for 1,2-disubstituted cyclopentane derivatives with phenyl and benzyl units
Source: Mol Divers. 2024 Jul 8;29(2):1305–21. doi: 10.1007/s11030-024-10911-y (PMC11909056; doi:10.1007/s11030-024-10911-y)

**Supplementary material**

**Investigation of cholinesterase and α-glucosidase enzyme activities, and molecular docking and dft studies for 1,2-disubstituted cyclopentane derivatives with phenyl and benzyl units**

Tekin Artunç^a^, Yasin Çetinkaya^a,*^, Parham Taslimi^b,*^, Abdullah Menzek^a,c,*^

*^a^Department of Chemistry, Faculty of Science, Atatürk University, 25240 Erzurum, Türkiye*

*^b^Department of Biotechnology, Faculty of Science, Bartin University, 74100 Bartin, Türkiye*

*^c^Department of Emergency Aid and Disaster Management, Faculty of Health Sciences,*

*Ardahan University, 75002 Ardahan, Türkiye*

**Contents** **Page**

**Table S1** Selected structural parameters of **10-13** S2

[**Table S2**](file:///C:\RJB%20Howard(ARTICAL)-YAYINLANACAK-GUNCEL\2017-HU-CEC10(MakaleRahimeSimsek)\shelx%20_geom_hbond_atom_site_label_D%20tablenum)  Natural and Mulliken charges on the atoms for **10** and **11**  S2

[**Table S3**](file:///C:\RJB%20Howard(ARTICAL)-YAYINLANACAK-GUNCEL\2017-HU-CEC10(MakaleRahimeSimsek)\shelx%20_geom_hbond_atom_site_label_D%20tablenum)  Natural and Mulliken charges on the atoms for **12** and **13**  S3

**Table S4**. Cartesian coordinates for the optimized structures of **10-13** S4-S9

^1^H and ^13^C NMR spectra of **10** S10

^1^H and ^13^C NMR spectra of **11** S11

^1^H and ^13^C NMR spectra of **12** S12

HMQC spectrum of **12** S13

^1^H and ^13^C NMR spectra of **13** S14

HRMS spectra of **10-13** S15

| **Table S1.** Selected structural parameters of **10-13** using the B3LYP/6-31+G(d,p) basis set.   \|  \| **10** \| **11** \|  \| **12** \| **13** \| \| --- \| --- \| --- \| --- \| --- \| --- \| \| *Bond lengths (Å)* \|  \|  \|  \|  \|  \| \| C3-C9 \| 1.494 \| 1.514 \| C3-C7 \| 1.513 \| 1.514 \| \| C9-C10 \| 1.485 \| 1.540 \| C7-C8 \| 1.543 \| 1.541 \| \| C10-C12 \| 1.355 \| 1.551 \| C8-C10 \| 1.556 \| 1.550 \| \| C12-C18 \| 1.481 \| 1.516 \| C10-C16 \| 1.520 \| 1.514 \| \| *Bond angles (^o^)* \|  \|  \|  \|  \|  \| \| C3-C9-C10 \| 120.1 \| 114.7 \| C3-C7-C8 \| 113.0 \| 114.0 \| \| C9-C10-C12 \| 128.4 \| 113.7 \| C7-C8-C10 \| 112.7 \| 113.1 \| \| C10-C12-C18 \| 126.6 \| 116.1 \| C8-C10-C16 \| 114.8 \| 115.1 \| \| C12-C18-C20 \| 121.2 \| 121.3 \| C10-C16-C18 \| 118.1 \| 121.3 \| \| *Dihedral angles (^o^)* \|  \|  \|  \|  \|  \| \| C4-C3-C9-O10 \| -41.6 \| -76.0 \| C4-C3-C7-C8 \| -173.5 \| -76.9 \| \| C3-C9-C10-C12 \| 135.6 \| -179.6 \| C3-C7-C8-C10 \| 163.6 \| 174.0 \| \| C9-C10-C12-C18 \| -7.3 \| -66.9 \| C7-C8-C10-C16 \| -68.1 \| -64.8 \| \| C10-C12-C18-C20 \| 126.9 \| 124.7 \| C8-C10-C16-C18 \| 115.2 \| 125.0 \| \| C4-C3-C9-C10 \| -41.6 \| -76.0 \|  \|  \|  \| \| C19-C18-C12-C10 \| -52.2 \| -55.5 \|  \|  \|  \| |
| --- | --- | --- | --- | --- | --- | --- | --- | --- | --- | --- | --- | --- | --- | --- | --- | --- | --- | --- | --- | --- | --- | --- | --- | --- | --- | --- | --- | --- | --- | --- | --- | --- | --- | --- | --- | --- | --- | --- | --- | --- | --- | --- | --- | --- | --- | --- | --- | --- | --- | --- | --- | --- | --- | --- | --- | --- | --- | --- | --- | --- | --- | --- | --- | --- | --- | --- | --- | --- | --- | --- | --- | --- | --- | --- | --- | --- | --- | --- | --- | --- | --- | --- | --- | --- | --- | --- | --- | --- | --- | --- | --- | --- | --- | --- | --- | --- | --- | --- | --- | --- | --- | --- | --- | --- | --- | --- | --- | --- |

[**Table S2.**](file:///C:\RJB%20Howard(ARTICAL)-YAYINLANACAK-GUNCEL\2017-HU-CEC10(MakaleRahimeSimsek)\shelx%20_geom_hbond_atom_site_label_D%20tablenum)  Natural and Mulliken charges on the atoms for compounds **10** and **11**.

| Atoms | **10** | | **11** | |
| --- | --- | --- | --- | --- |
|  | Natural charges | Mulliken  charges | Natural charges | Mulliken  charges |
| C1 | -0.33368 | 0.052041 | -0.33085 | 0.127033 |
| C2 | -0.18836 | -0.430534 | -0.22173 | -0.326295 |
| C3 | -0.13142 | 0.605670 | -0.08105 | 0.769081 |
| C4 | 0.31276 | -0.303549 | 0.29705 | -0.564757 |
| C5 | 0.20966 | -0.557262 | 0.21736 | -0.494808 |
| C6 | 0.29515 | 0.230106 | 0.27927 | 0.263333 |
| C9 | 0.21554 | -0.171475 | -0.46681 | -0.036436 |
| C10 | -0.06715 | 0.620369 | -0.25612 | -0.141436 |
| C11 | -0.47479 | -0.659298 | -0.46747 | -0.264840 |
| C12 | -0.00085 | -0.235293 | -0.28308 | 0.012034 |
| C13 | -0.46890 | -0.634620 | -0.48081 | -0.501799 |
| C16 | -0.47822 | 0.247367 | -0.45544 | -0.331789 |
| C18 | -0.12713 | 0.693696 | -0.07663 | 0.107743 |
| C19 | -0.19519 | -1.038177 | -0.21712 | -0.211300 |
| C20 | 0.29934 | 0.070550 | 0.29011 | -0.052992 |
| C21 | -0.32953 | -0.276808 | -0.32495 | 0.152558 |
| C23 | 0.23148 | -0.312841 | 0.22913 | -0.401805 |
| C24 | 0.29557 | 0.405090 | 0.28159 | 0.348224 |
| O26 | -0.55843 | -0.228935 | -0.56109 | -0.296764 |
| O27 | -0.56617 | -0.404694 | -0.56631 | -0.403235 |
| O28 | -0.54225 | -0.384840 | -0.54691 | -0.389452 |
| O29 | -0.57107 | -0.281012 | -0.57325 | -0.284388 |
| O30 | -0.57408 | -0.339239 | -0.57180 | -0.341660 |
| O31 | -0.53293 | -0.354730 | -0.53961 | -0.378913 |
| C32 | -0.32190 | -0.090254 | -0.32204 | -0.141963 |
| C36 | -0.32598 | -0.162743 | -0.32636 | -0.165237 |
| C40 | -0.33251 | -0.123358 | -0.33106 | -0.129417 |
| C44 | -0.32683 | -0.096257 | -0.32526 | -0.087825 |
| C48 | -0.33215 | -0.172219 | -0.32236 | -0.134984 |
| C52 | -0.33294 | -0.144774 | -0.33167 | -0.140011 |
| N59 | -0.25729 | 0.152556 |  |  |
| N60 | -0.46622 | -0.093110 |  |  |
| C62 | 0.51095 | 0.350646 |  |  |
| O63 | -0.62177 | -0.424395 |  |  |

[**Table S3.**](file:///C:\RJB%20Howard(ARTICAL)-YAYINLANACAK-GUNCEL\2017-HU-CEC10(MakaleRahimeSimsek)\shelx%20_geom_hbond_atom_site_label_D%20tablenum)  Natural and Mulliken charges on the atoms for compounds **12** and **13**.

| Atoms | **12** | | **13** | |
| --- | --- | --- | --- | --- |
|  | Natural charges | Mulliken  charges | Natural charges | Mulliken  charges |
| C1 | -0.19510 | -0.084063 | -0.18604 | -0.096752 |
| C2 | -0.11735 | -0.065559 | -0.13030 | -0.269237 |
| C3 | -0.07798 | 0.332913 | -0.06829 | 0.431620 |
| C4 | 0.30962 | 0.127604 | 0.29867 | 0.045050 |
| C5 | 0.22644 | 0.187761 | 0.24963 | -0.014447 |
| C6 | 0.26447 | -0.423464 | 0.25638 | -0.341252 |
| C7 | -0.47621 | -0.443676 | -0.47808 | -0.141985 |
| C8 | -0.25555 | -0.165950 | -0.24667 | -0.320923 |
| C9 | -0.46727 | -0.107837 | -0.47202 | -0.181031 |
| C10 | -0.28080 | -0.532427 | -0.27826 | 0.079551 |
| C11 | -0.47809 | -0.595497 | -0.48077 | -0.522540 |
| C14 | -0.46464 | -0.311939 | -0.46013 | -0.344127 |
| C16 | -0.04973 | 0.745431 | -0.03736 | -0.022663 |
| C17 | -0.12684 | -0.393000 | -0.24269 | 0.392952 |
| C18 | 0.28666 | -0.075047 | 0.27705 | -0.111424 |
| C19 | -0.18396 | 0.120182 | -0.15572 | 0.162884 |
| C20 | 0.24655 | 0.301329 | 0.24679 | 0.021354 |
| C21 | 0.26696 | -0.723617 | 0.25170 | -0.741383 |
| O22 | 0.55014 | -0.266350 | -0.55932 | -0.288013 |
| O23 | -0.57143 | -0.418008 | -0.56943 | -0.402623 |
| O24 | -0.57139 | -0.325764 | -0.57147 | -0.333066 |
| O25 | -0.57390 | -0.255276 | -0.57248 | -0.296990 |
| O26 | -0.57744 | -0.368832 | -0.57900 | -0.359274 |
| O27 | -0.56302 | -0.266382 | -0.56734 | -0.299425 |
| C28 | -0.32089 | -0.097386 | -0.32524 | -0.138871 |
| C32 | -0.32851 | -0.119557 | -0.33041 | -0.124158 |
| C36 | -0.32884 | -0.143962 | -0.32874 | -0.145225 |
| C40 | -0.32649 | -0.082134 | -0.32595 | -0.091076 |
| C44 | -0.32606 | -0.115020 | -0.32592 | -0.100285 |
| C48 | -0.32741 | -0.126353 | -0.32633 | -0.119320 |
| Br59 | 0.09464 | 0.134699 | 0.09364 | 0.133642 |
| Br60 | 0.10556 | 0.120719 | 0.10790 | 0.127908 |
| Br61 | 0.10653 | 0.104228 | 0.06750 | 0.120949 |
| Br62 | 0.10714 | 0.174129 |  |  |

**Table S4.** Cartesian coordinates for the optimized structures of **10-13**

**Compound 10**


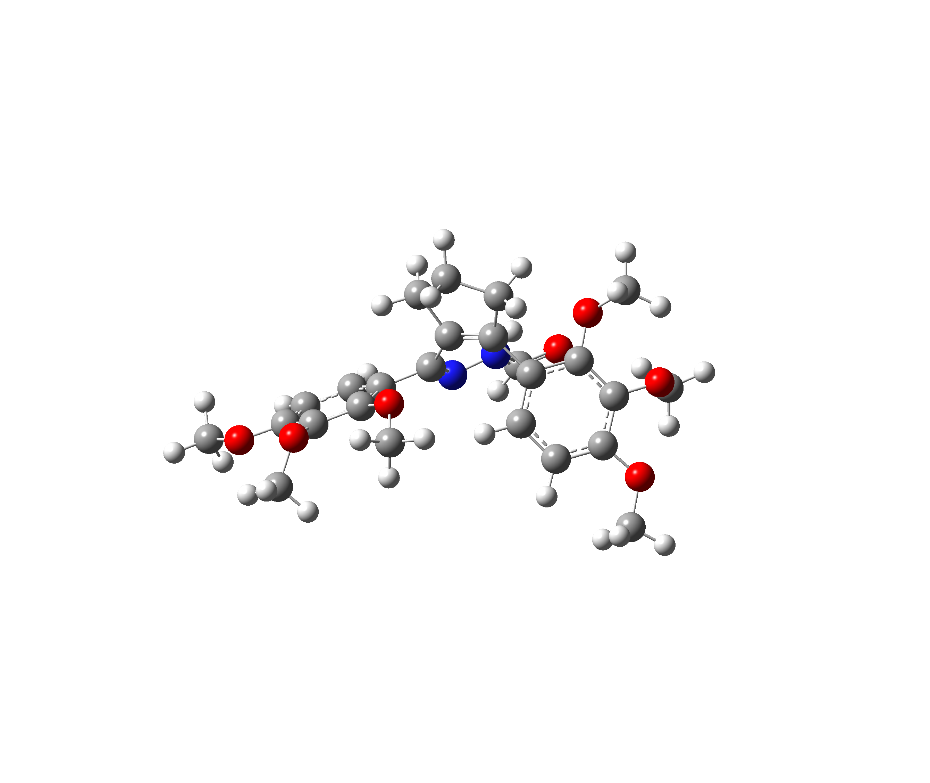


----------------------------------------------------

Center Coordinates (Angstroms)

Number X Y Z

----------------------------------------------------

1 -4.361625 0.328270 -1.941393

2 -3.013845 0.669998 -1.855105

3 -2.266484 0.472710 -0.688835

4 -2.919241 -0.107476 0.427704

5 -4.284898 -0.443898 0.363979

6 -5.008469 -0.212345 -0.827530

7 -4.900830 0.514816 -2.861615

8 -2.516234 1.105085 -2.714045

9 -0.824954 0.866500 -0.675218

10 -0.246610 1.535615 0.518150

11 -0.970575 2.709164 1.163789

12 0.934220 1.285776 1.133233

13 -0.213318 2.910925 2.495082

14 -2.036911 2.512294 1.311062

15 -0.900531 3.594576 0.513877

16 1.178305 2.281897 2.259119

17 -0.732988 2.363876 3.289314

18 1.865044 0.171873 0.839211

19 1.414890 -1.153197 0.810069

20 3.229734 0.412333 0.561494

21 2.273724 -2.218278 0.532485

22 0.369593 -1.351375 1.019606

23 4.096191 -0.639273 0.230235

24 3.619341 -1.968074 0.242690

25 1.886567 -3.229867 0.538379

26 -2.174622 -0.260046 1.564368

27 -4.917015 -0.878003 1.506079

28 -6.340342 -0.514392 -0.782704

29 3.639820 1.721794 0.483951

30 5.403132 -0.370657 -0.091708

31 4.540142 -2.926701 -0.065536

32 -2.309635 -1.425547 2.384505

33 -3.148282 -1.332345 3.076212

34 -2.436035 -2.323930 1.769009

35 -1.372438 -1.499216 2.941241

36 -5.510745 -2.183890 1.478700

37 -6.339085 -2.228817 0.768687

38 -4.762061 -2.944669 1.222312

39 -5.879787 -2.363527 2.490512

40 -7.134271 -0.253193 -1.935615

41 -8.149065 -0.550264 -1.668197

42 -7.122388 0.812645 -2.192907

43 -6.795563 -0.842788 -2.796234

44 4.726114 2.131657 1.330463

45 4.461367 1.983341 2.384833

46 5.639498 1.582658 1.094453

47 4.864890 3.196802 1.136123

48 5.686893 -0.395265 -1.506012

49 5.578534 -1.411108 -1.899548

50 5.025681 0.291506 -2.044723

51 6.725200 -0.073422 -1.606768

52 4.120057 -4.285420 -0.084803

53 5.006694 -4.865140 -0.344475

54 3.752971 -4.603734 0.898936

55 3.340305 -4.453105 -0.838376

56 -0.159448 3.960946 2.798566

57 1.583211 1.796325 3.154776

58 1.916646 3.029528 1.942825

59 -0.217303 0.697370 -1.810864

60 1.053163 1.152970 -1.983136

61 1.551123 1.655421 -1.249160

62 1.696958 0.893773 -3.161486

63 2.846097 1.237823 -3.399399

64 1.059841 0.334762 -3.866552

----------------------------------------------------

**
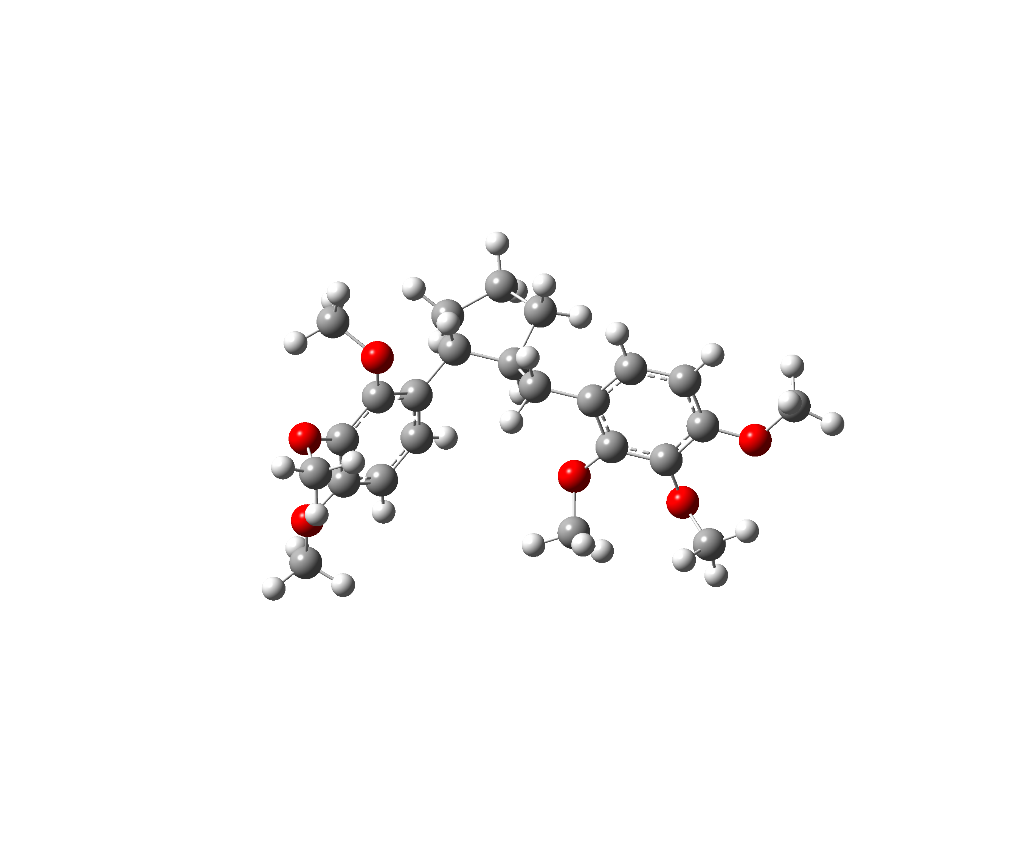
 Compound 11**

--------------------------------------------------

Center Coordinates (Angstroms)

Number X Y Z

--------------------------------------------------

1 4.496108 1.112672 1.244730

2 3.130424 1.416033 1.244454

3 2.189761 0.611666 0.603337

4 2.654532 -0.554517 -0.045844

5 4.022739 -0.878706 -0.066757

6 4.948675 -0.024829 0.575728

7 5.190069 1.771842 1.751484

8 2.791564 2.306545 1.766755

9 0.718204 0.967338 0.600238

10 0.199924 1.546630 -0.729291

11 0.852896 2.873796 -1.161410

12 -1.307706 1.911978 -0.704040

13 -0.129055 3.504806 -2.184702

14 1.858680 2.734647 -1.569050

15 0.953459 3.526072 -0.283052

16 -1.480181 2.760249 -1.991389

17 0.236209 3.380873 -3.209836

18 -2.274129 0.751260 -0.577559

19 -2.257880 -0.318636 -1.478848

20 -3.233539 0.706912 0.455217

21 -3.149119 -1.390611 -1.387209

22 -1.526662 -0.323084 -2.281915

23 -4.131066 -0.366912 0.575650

24 -4.097208 -1.419704 -0.359542

25 -3.098491 -2.189232 -2.117708

26 1.706824 -1.296354 -0.705158

27 4.442077 -1.954694 -0.815437

28 6.267430 -0.383216 0.464513

29 -3.255902 1.705867 1.406059

30 -5.077060 -0.345834 1.574471

31 -5.017197 -2.415486 -0.171357

32 1.699789 -2.721194 -0.580283

33 2.407814 -3.185508 -1.269915

34 1.932556 -3.024052 0.447966

35 0.681132 -3.032992 -0.822767

36 5.108979 -3.017130 -0.120476

37 6.061386 -2.682635 0.296389

38 4.473047 -3.414048 0.681627

39 5.283549 -3.796732 -0.865072

40 7.248496 0.480766 1.023372

41 8.212106 0.024504 0.791997

42 7.201731 1.480393 0.573905

43 7.137272 0.564339 2.111884

44 -4.371645 2.603684 1.332201

45 -4.374506 3.135037 0.371638

46 -5.316217 2.068203 1.464479

47 -4.235915 3.321874 2.143671

48 -4.810799 -1.216774 2.681607

49 -4.783091 -2.262500 2.358196

50 -3.864016 -0.946506 3.164655

51 -5.633727 -1.073409 3.384895

52 -5.057816 -3.479065 -1.113264

53 -5.867571 -4.133426 -0.787335

54 -5.273163 -3.109933 -2.123860

55 -4.116248 -4.043085 -1.124474

56 -0.232650 4.582394 -2.019380

57 -1.678049 2.098284 -2.842345

58 -2.335271 3.440946 -1.918145

59 0.126792 0.076829 0.841793

60 0.533813 1.700114 1.396636

61 0.367485 0.798410 -1.515391

62 -1.464782 2.565288 0.162034

--------------------------------------------------

**Compound 12**

**
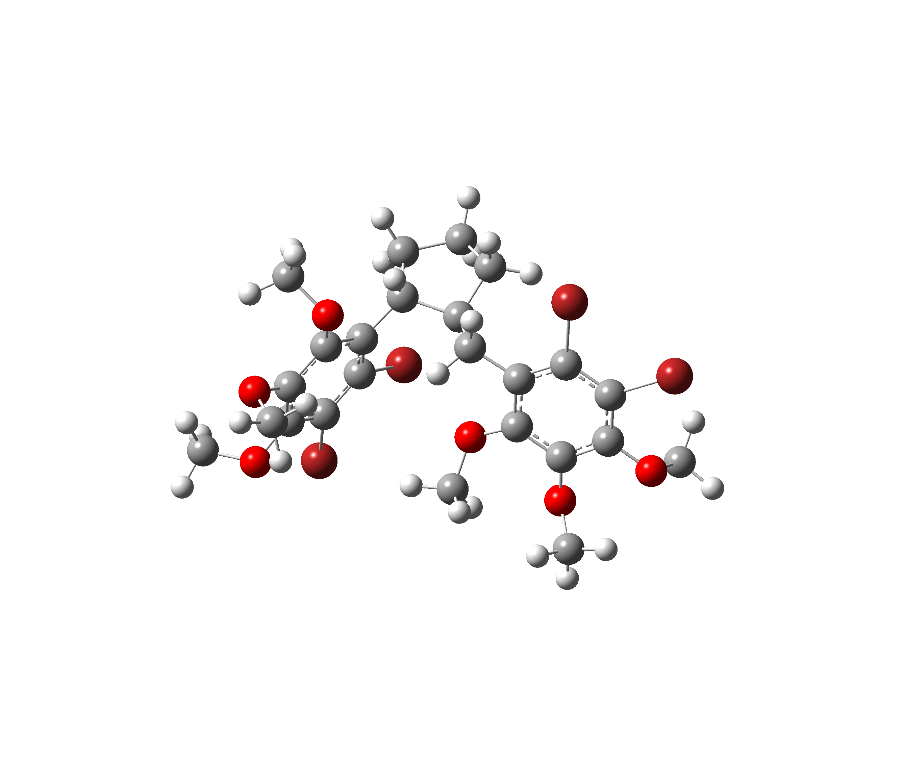
**

---------------------------------------------------

Center Coordinates (Angstroms)

Number X Y Z

---------------------------------------------------

1 4.266699 0.275547 -0.432775

2 3.248350 -0.685159 -0.370082

3 1.897382 -0.319066 -0.379663

4 1.573803 1.057886 -0.473472

5 2.587054 2.031140 -0.521448

6 3.934877 1.631612 -0.491422

7 0.755941 -1.307606 -0.281812

8 0.142463 -1.382103 1.132250

9 0.911373 -2.197044 2.186074

10 -1.254666 -2.066174 1.146313

11 -0.111635 -2.365733 3.326674

12 1.837867 -1.711170 2.508856

13 1.186698 -3.176241 1.771869

14 -1.489810 -2.515621 2.624761

15 -0.111552 -1.467983 3.955725

16 -2.358786 -1.277897 0.461220

17 -2.787963 0.009862 0.840243

18 -2.948323 -1.827572 -0.697997

19 -3.730853 0.720051 0.086436

20 -3.904619 -1.130097 -1.450999

21 -4.298403 0.155466 -1.064018

22 0.240584 1.341104 -0.429262

23 2.267464 3.368369 -0.501155

24 4.915228 2.589783 -0.542208

25 -2.537655 -3.058351 -1.159936

26 -4.484341 -1.715201 -2.550110

27 -5.163806 0.872813 -1.841688

28 -0.323039 2.469665 -1.111105

29 -0.143110 3.395601 -0.564142

30 0.080850 2.549705 -2.126592

31 -1.393767 2.268033 -1.160949

32 2.608847 4.125296 -1.676225

33 3.681761 4.079104 -1.875107

34 2.052396 3.753872 -2.545621

35 2.309631 5.153574 -1.465032

36 5.269419 3.154553 0.733152

37 6.055177 3.883882 0.529851

38 4.406053 3.650836 1.188674

39 5.655243 2.379127 1.404017

40 -3.442053 -4.142565 -0.894083

41 -3.567793 -4.281650 0.186763

42 -4.414217 -3.962236 -1.362922

43 -2.980952 -5.032650 -1.326044

44 -3.759276 -1.528650 -3.778927

45 -3.690197 -0.463214 -4.024426

46 -2.758417 -1.966732 -3.703978

47 -4.332515 -2.046801 -4.549695

48 -6.551182 0.515335 -1.713389

49 -7.091108 1.168532 -2.400606

50 -6.707682 -0.531046 -1.991913

51 -6.899423 0.693833 -0.689598

52 0.121336 -3.214058 3.978310

53 -2.258552 -1.922008 3.124360

54 -1.833929 -3.555742 2.640122

55 -0.023976 -0.982043 -0.976882

56 1.084182 -2.299871 -0.599566

57 0.042080 -0.361226 1.506926

58 -1.140346 -2.975723 0.551626

59 3.721501 -2.530595 -0.293136

60 6.105258 -0.186863 -0.414065

61 -2.130814 0.822066 2.438207

62 -4.338207 2.449088 0.583640

---------------------------------------------------

**Compound 13**

**
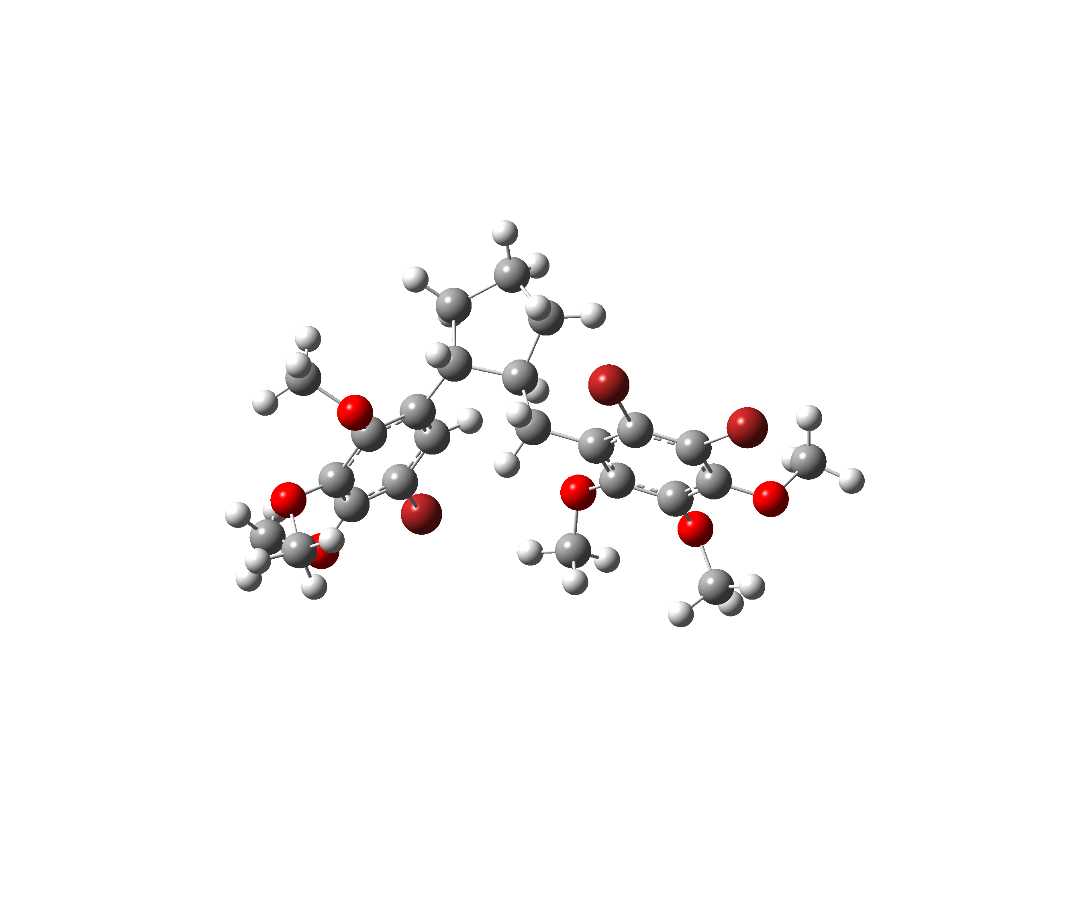
**

---------------------------------------------------

Center Coordinates (Angstroms)

Number X Y Z

---------------------------------------------------

1 4.116401 0.122947 -0.367530

2 2.974547 -0.686800 -0.305629

3 1.694991 -0.132856 -0.159411

4 1.582927 1.274905 -0.086895

5 2.720314 2.097923 -0.146615

6 3.991829 1.512610 -0.269720

7 0.428727 -0.957860 -0.071276

8 -0.133770 -1.091540 1.357143

9 0.695414 -1.913147 2.360417

10 -1.505730 -1.811649 1.404647

11 -0.283697 -2.202246 3.525611

12 1.603606 -1.396913 2.687213

13 1.011925 -2.851334 1.889051

14 -1.714100 -2.078693 2.920725

15 -0.149624 -1.473800 4.332673

16 -2.639293 -1.078205 0.718831

17 -2.931835 0.252213 1.048161

18 -3.407141 -1.693768 -0.286357

19 -3.941556 0.951214 0.397457

20 -4.428121 -0.984825 -0.946403

21 -4.709513 0.348243 -0.604766

22 0.323518 1.767215 0.115096

23 2.588180 3.454259 0.021724

24 5.099152 2.320601 -0.321619

25 -3.109010 -2.976208 -0.689185

26 -5.182274 -1.611950 -1.910925

27 -5.660216 1.050068 -1.297259

28 -0.151020 2.874665 -0.665172

29 0.207751 3.823158 -0.261850

30 0.160828 2.770128 -1.711078

31 -1.239693 2.830969 -0.605515

32 2.973788 4.281797 -1.091429

33 4.021751 4.124021 -1.354574

34 2.336402 4.075016 -1.959624

35 2.818592 5.310967 -0.762806

36 5.646214 2.674311 0.961347

37 4.915024 3.238254 1.550253

38 5.959237 1.777577 1.507206

39 6.518179 3.296615 0.753806

40 -4.059675 -3.982612 -0.309138

41 -4.128023 -4.052618 0.783808

42 -5.045587 -3.769400 -0.732208

43 -3.679013 -4.923631 -0.710823

44 -4.735762 -1.379394 -3.258120

45 -4.785438 -0.312771 -3.501104

46 -3.713688 -1.751275 -3.391551

47 -5.417031 -1.936703 -3.903737

48 -7.015501 0.811552 -0.882961

49 -7.640875 1.425700 -1.533041

50 -7.276736 -0.244642 -1.003713

51 -7.157878 1.120582 0.159098

52 -0.105507 -3.189782 3.962853

53 -2.255187 -1.242122 3.376473

54 -2.322493 -2.973807 3.087694

55 -0.333806 -0.473126 -0.689114

56 0.603154 -1.952267 -0.487652

57 -0.259272 -0.079518 1.763645

58 -1.384443 -2.774410 0.896088

59 3.176383 -2.576752 -0.459288

60 5.859635 -0.593749 -0.568800

61 -4.304443 2.757996 0.892266

62 -2.352752 0.758229 1.812126

---------------------------------------------------

^1^H NMR and ^13^C NMR spectra of compounds **10-13**.

**Compound 10**

**Compound 11**

**Compound 12**

HMQC spectrum of compound **12**

**Compound 13**

**
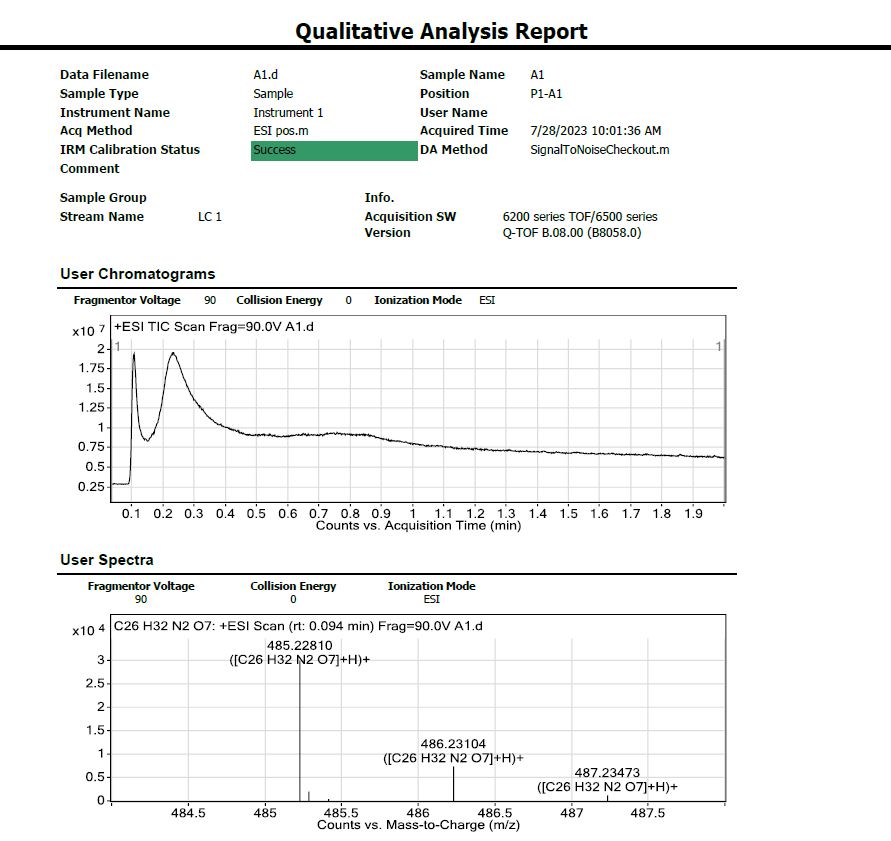
HRMS spectra of 10-13**

HRMS spectrum of **10
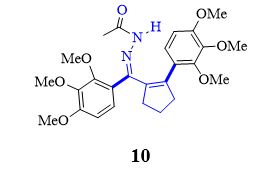
**

HRMS spectrum of **11**


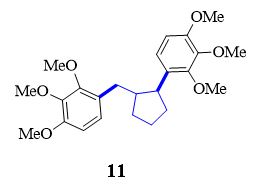
**
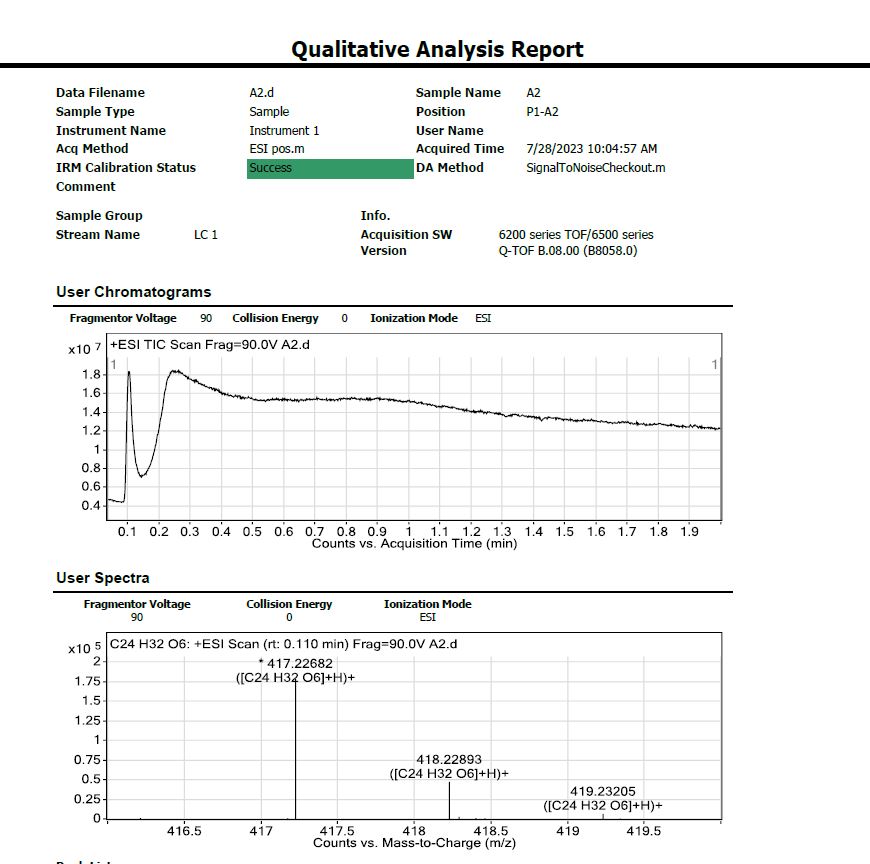
**

HRMS spectrum **of 12**


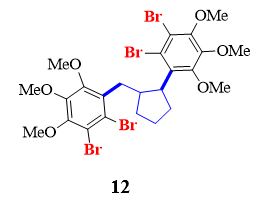

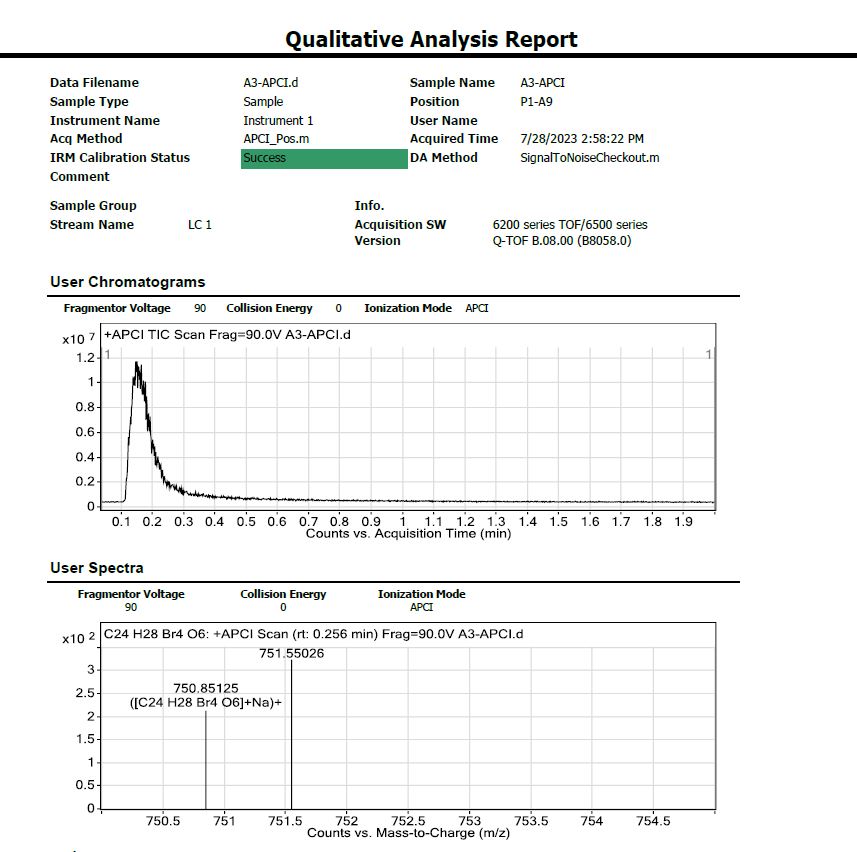


HRMS spectrum **of 13**


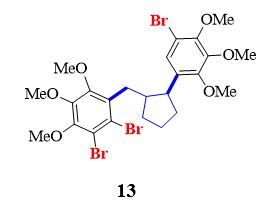

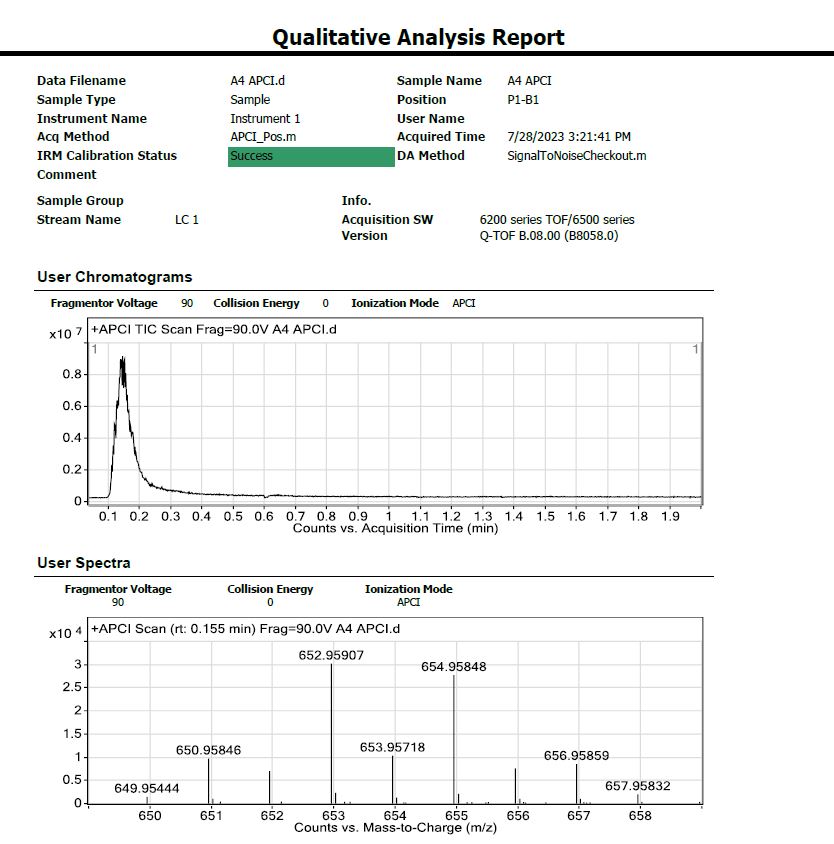

Supplement: Supplementary file 1 — Supplementary file1 (DOCX 27102 KB) [file 11030_2024_10911_MOESM1_ESM.docx]
